# Identification of nutrition factors in the metabolic syndrome and its progression over time in older adults: analysis of the TUDA cohort

**DOI:** 10.1186/s13098-024-01367-z

**Published:** 2024-06-08

**Authors:** Oonagh C. Lyons, Maeve A. Kerr, Mary A. T. Flynn, Leane Hoey, Catherine F. Hughes, Aoife Caffrey, Eamon Laird, Katie Moore, Kirsty M. Porter, Conal Cunningham, Kevin McCarroll, Anne M. Molloy, Fergal Tracey, Maurice O’Kane, J. J. Strain, Mary Ward, Helene McNulty

**Affiliations:** 1 Nutrition Innovation Centre for Food and Health (NICHE), School of Biomedical Sciences, Ulster University, Cromore Road, Coleraine, BT52 1SA Northern Ireland, UK; 2 Food Safety Authority of Ireland, Dublin, Ireland; 3 School of Medicine, Trinity College Dublin, Dublin, Ireland; 4 Mercer’s Institute for Research on Ageing, St James’s Hospital, Dublin, Ireland; 5 Causeway Hospital, Northern Health and Social Care Trust, Coleraine, Northern Ireland, UK; 6 Clinical Chemistry Laboratory, Western Health and Social Care Trust, Altnagelvin Hospital, Londonderry, Northern Ireland, UK

**Keywords:** Metabolic syndrome, Older adults, Nutrition-related factors, Protein quality

## Abstract

**Background:**

Nutrition is recognized as playing an important role in the metabolic syndrome (MetS), but the dietary components involved are unclear. We aimed to investigate nutrition factors in relation to MetS and its progression in older adults over a follow-up period of 5.4 years.

**Methods:**

Community-dwelling adults (≥ 60y) from the Trinity-Ulster-Department-of-Agriculture study, sampled at baseline (2008–12) and follow-up (2014–18; *n* 953), were classified as ‘with MetS’ by having three or more of: waist circumference (≥ 102 cm, males; ≥ 88 cm, females); HDL-cholesterol (< 1.0 mmol/L, males; < 1.3 mmol/L, females); triglycerides (≥ 1.7 mmol/L); blood pressure (systolic ≥ 130 and/or diastolic ≥ 85 mmHg); and hemoglobin A1c (≥ 39 mmol/mol).

**Results:**

MetS was identified in 67% of participants, increasing to 74% at follow-up. Predictors at baseline for the development of metabolic syndrome (MetS) at follow-up were higher waist circumference (odds ratio [95%CI]; 1.06 [1.01–1.11]), but not BMI, and increased triglyceride concentrations (2.01 [1.29–3.16]). In dietary analysis (at follow-up), higher protein (g/kg bodyweight/day) and monounsaturated fatty acid (g/day) intakes were each associated with lower risk of MetS (0.06 [0.02–0.20] and 0.88 [0.78–1.00], respectively), whilst higher protein was also associated with lower abdominal obesity (0.10 [0.02–0.51]) and hypertension (0.22 [0.00–0.80]). Furthermore, participants with, compared to without, MetS consumed less high-quality protein foods (*P* = 0.006) and more low-quality protein foods (*P* < 0.001), as defined by the protein digestibility-corrected amino acid score.

**Conclusions:**

Dietary interventions targeting protein quantity and quality may have specific benefits in preventing or delaying the progression of MetS in at-risk older people, but this requires investigation in the form of randomized trials.

**Supplementary Information:**

The online version contains supplementary material available at 10.1186/s13098-024-01367-z.

## Background

The metabolic syndrome (MetS), as originally described by Reaven in 1988 [1], refers to a clustering of abnormal metabolic components, namely, central obesity, hypertension, dyslipidemia and insulin resistance, leading to disease in aging. MetS is a prothrombotic, proinflammatory state [2] widely reported to increase the risk of cardiovascular disease (CVD) by up to two-fold and type 2 diabetes mellitus (T2DM) by five-fold, both major causes of morbidity and mortality [3, 4]. While the underlying pathophysiology of MetS is complex and not fully understood, it is generally accepted that insulin resistance, hormonal activation and inflammation contribute significantly to the progression of MetS and the concomitant disease end points in aging, CVD and T2DM [3, 5]. Insulin resistance causes an increase in circulating free fatty acids, ultimately leading to hyperinsulinemia and contributing to hypertension and reduced HDL cholesterol [5]. Increased leptin and reduced adiponectin concentrations, which may occur as a result of obesity [5], are associated with an increased risk of CVD and inflammation [5]. The latter plays an important role in the pathogenesis of CVD and various inflammatory markers are reported to be elevated in adults with MetS [5].

Various definitions of MetS have been proposed by the World Health Organization (WHO) [6], the National Cholesterol Education Program Adult Treatment Panel III (ATPIII) [7] and the International Diabetes Federation (IDF) [8]. However, in 2009 a harmonized definition, the Joint Interim Statement (JIS), was developed comprising a single set of cut-offs for all components of MetS, except for waist circumference where national cut-offs can be used [4]. The JIS identifies MetS as having three or more of the following criteria: abdominal obesity, elevated triglycerides, reduced HDL cholesterol, elevated blood pressure and impaired fasting blood glucose [4].

Globally, MetS is estimated to affect 25% of the world’s adult population [9, 10] and typically increases with age [9, 11, 12], along with the prevalence of other chronic conditions such as CVD, T2DM and hypertension [11, 13]. Concurrently, populations worldwide are aging, with estimations that by 2050 one in six people will be aged 65 years or older [14]. Furthermore, the global obesity epidemic is contributing to an increased prevalence of MetS among older adults [11]. MetS is thus a major public health concern, affecting quality of life for a considerable, and growing, proportion of the world’s population and placing a significant burden on economic and health care systems worldwide [15, 16].

Lifestyle and environmental factors, including excess dietary energy intake and physical inactivity, along with the consequent abdominal obesity, have been identified as major contributors to the development of MetS [2, 3]. Previous studies have reported that body mass index (BMI) [17, 18], waist circumference [18, 19] and socioeconomic status [18, 20] play important roles in the onset of MetS, whilst in older adults, age, sex, education and physical inactivity are associated with MetS risk [21]. Thus, interventions involving weight loss and related lifestyle changes have resulted in significant reductions in MetS components [10, 22]. Some studies have focused on dietary patterns or specific dietary components [10, 23] or the role of dietary macronutrients [24–26] in relation to MetS. However, the relative contribution of specific dietary components in the development and progression of MetS remains unclear owing to the limited evidence base.

A better understanding of the nutrition-related factors that contribute to the progression of MetS and its components may help to inform effective nutrition intervention strategies aimed at preventing MetS and associated pathologies in older people. Therefore, this study aimed to investigate nutrition factors in relation to MetS and its progression over a minimum follow-up period of 5 years.

## Methods

### Study design and sample

This observational study involved secondary analysis of data from the Trinity-Ulster-Department of Agriculture (TUDA) cohort (ClinicalTrials.gov identifier NCT02664584). As described in detail elsewhere [27], 5186 community-dwelling adults aged ≥ 60 years were recruited between 2008 and 2012 from General Practice or hospital outpatient clinics in Northern Ireland (UK) and the Republic of Ireland via standardized protocols. The TUDA study initially aimed to investigate the role of nutrition and lifestyle factors in the development of three common diseases of ageing, namely, dementia, osteoporosis, and cardiovascular disease. Briefly, the inclusion criteria for the TUDA study were: born on the island of Ireland, aged ≥ 60 years, and without an existing diagnosis of dementia. Participants recruited in Northern Ireland had been diagnosed with hypertension (hypertensive sub-cohort, sub-cohort 1) and were recruited from General Practices in the catchment areas of the Western and Northern Health and Social Care Trusts. Participants recruited from the Republic of Ireland had been referred to outpatient bone clinics (bone sub-cohort, sub-cohort 2; majority had osteopenia/osteoporosis, but some were found to have normal bone health following a scan) or memory (cognitive sub-cohort, sub-cohort 3) clinics at St. James’s Hospital, Dublin.

The current study also includes analysis of data from approximately 20% of the original TUDA participants who were re-sampled after a minimum of 5 years following initial sampling (median follow-up of 5.4 years) for the full range of biomarkers and health measures and additionally included comprehensive dietary intake data. The exclusion criteria for follow-up were as follows: those aged < 65 years, a recorded Folstein Mini-Mental State Examination (MMSE) score < 21 (at initial sampling), on vitamin B12 injections, those recruited from memory clinics (sub-cohort 3) and those who were uncontactable, unable or unwilling to participate at follow-up.

Ethical approval was granted by the Office for Research Ethics Committees Northern Ireland (ORECNI; reference 08/NIRO3/113), with corresponding approvals from the Northern and Western Health and Social Care Trusts in Northern Ireland, and the Research Ethics Committee of St James Hospital and The Adelaide and Meath Hospital in Dublin. All participants provided written informed consent at the time of recruitment.

### Blood sampling and laboratory analysis

A non-fasting blood sample (50 ml) was obtained from each participant and processed within 4 h of collection. Analysis for routine clinical blood biochemistry profile and hemoglobin A1c (HbA1c) was performed at the time of blood collection. HbA1c measurement was performed in participating hospital laboratories on the Bio-Rad Variant II Turbo analyzer (Bio-Rad Laboratory Inc., Hercules, CA) which is traceable to the International Federation for Clinical Chemistry reference method; results were reported in units of mmol/mol.

Serum C-reactive protein (CRP) concentrations were measured using sandwich immunoassay with Meso Scale Discovery (MSD) V-PLEX Vascular Injury Panel 2 (human) kit (Meso Scale Diagnostics, Maryland, USA). Serum concentrations of IL-10, IL-6 and TNF-α were measured using the MSD V-PLEX Pro-inflammatory Panel 1 (human) kit (Meso Scale Diagnostics, Maryland, USA). The inter-assay CV were 4.7%, 10.7%, 7.9% and 8.8% for CRP, IL-10, IL-6 and TNF-α, respectively. The kits were conducted in accordance with the manufacturer’s instructions and all samples were run in duplicates.

### Dietary assessment

Dietary intake data was collected only from the TUDA follow-up study (2014–2018). Dietary intake was collected using an unweighed 4-day food diary (over 4 consecutive days, including Saturday and Sunday, to account for the known variation in day-to-day intake) in combination with a researcher-assisted food frequency questionnaire (FFQ) designed to collect detailed information on the frequency of specific foods of interest, an approach that has been previously validated against biomarker data at our center [28]. Each participant received oral and written instructions on how to complete the 4-day food diary and FFQ. Any queries on reported information or discrepancies between the two dietary records were discussed with the participant within one week of collection to enhance the accuracy of information regarding usual dietary intake. Food portion sizes were estimated by the participant using household measures and quantified using published food portion size data available in *Nutritics* (Version 5.76; Research Edition, Dublin, Ireland). Mean daily energy and macronutrient intakes were calculated using *Nutritics* nutrition analysis software. Food diaries were available for 84% (*n* 803) of the follow-up cohort.

The protein digestibility-corrected amino acid score (PDCAAS) was used to assess protein quality [29]. The PDCAAS relates the essential amino acid content of a foodstuff to a reference amino acid profile, after applying a correction term for protein digestibility. A PDCAAS below 100 indicates that at least one amino acid is limiting in the food or diet, whereas a score of 100 indicates no limiting amino acid in the food or diet [29]. For the purposes of this study, a previous review of foods commonly eaten by older adults in Ireland [30] was used to assign a PDCAAS to the foods providing protein as reported in the 4-day food diaries. Using the PDCAAS, these foods were then assigned to a protein quality category; category 1 (PDCAAS > 95), category 2 (PDCASS 80–90), category 3 (PDCAAS 60–70), or category 4 (PDCAAS < 35).

Basal metabolic rate (BMR) of participants was calculated from standard equations [31] using body weight (kg) and height (m). The BMR was multiplied by a physical activity level (PAL) of 1.61 from the UK Scientific Advisory Committee on Nutrition [32] to calculate the estimated energy requirements (EER) for each participant. Potential misreporting was estimated by calculating the percentage difference between reported energy intake (EI) and estimated energy requirements (EER) using the following equation as described by Kelly and colleagues [33]: (EI–EER)/EER*100 = Percentage of misreporting of energy needs (%EER). Potential mis-reporters were not excluded from analysis.

### Health, lifestyle, anthropometric and biophysical measures

As previously reported [34], health and lifestyle information were gathered using a researcher-assisted questionnaire. Anthropometric measurements (including weight, height, waist, and hip) were recorded. Blood pressure (BP) measurements were taken in accordance with standard operating procedures and clinic guidelines using an A&d ua-787 digital blood pressure monitor (Cardiac Services, Belfast, UK). Participants were seated with both feet flat on the floor and two BP measurements were taken in the reference arm after a 5 min rest period to calculate a mean BP value. If there was > 5 mmHg difference in BP additional measurements were taken and the mean of the two BP measurements in closest agreement was used. The Timed Up-and-Go (TUG) test and the Physical Self-Maintenance Scale (PSMS) were used to assess functional mobility and general ability of participants. The TUG test measured the time taken to stand up from seated in a chair, walk three meters, turn around and walk back to return to the original seated position [35]. The PSMS is a questionnaire which assigns scores to the participants highest level of functioning for activities of daily living, the higher the total score the more independent the participant [36]. Physical activity was reported as yes/no in the last two weeks. Area-based socioeconomic deprivation score was measured by adopting a novel cross-jurisdictional approach whereby geo-referenced address-based information was used to map and link participants to official socioeconomic indicators of deprivation within Northern Ireland (UK) and the Republic of Ireland, as previously described in detail elsewhere [27]. Deprivation scores were categorized into quintiles (Q1–5), with Q1 being the 20% least deprived category, and Q5 the 20% most deprived category.

### Metabolic syndrome categorization

In line with the JIS definition [4], participants were deemed to have MetS if they met at least three of the following criteria: waist circumference of ≥ 102 cm or ≥ 88 cm, for males and females, respectively [37]; elevated blood pressure of systolic ≥ 130 and/or diastolic ≥ 85 mmHg; HbA1c of ≥ 39 mmol/mol which was used as a surrogate marker for elevated fasting blood glucose [38]; reduced HDL cholesterol of < 1.0 mmol/L (< 40 mg/dL) for males and < 1.3 mmol/L (< 50 mg/dL) for females; and elevated triglycerides of ≥ 1.7 mmol/L (≥ 150 mg/dL). Usage of anti-hypertensive, diabetic and lipid-lowering (including statins) drugs were also considered as alternative indicators for having MetS [4].

### Statistical analysis

Statistical analysis was performed using SPSS software (Version 25.0. Armonk, NY: IBM Corp). For comparison between the same participants at both timepoints, continuous variables were analyzed using paired samples t-tests on log-transformed data and categorical variables analyzed using McNemar’s test. Chi-square was used to assess the differences in the proportion of participants affected by MetS and its components at baseline and follow-up. Binary logistic regression analysis was used to identify baseline predictors of MetS and its components at follow-up. As drug use will affect the development of MetS and its components, the following adjustments were made in this analysis: anti-hypertensive, diabetic and lipid-lowering drug use when identifying predictors of MetS; anti-hypertensive drug use when identifying predictors of hypertension; diabetic drug use when identifying predictors of hyperglycemia; and lipid-lowering drug use when identifying predictors of dyslipidemia. We also adjusted for the time interval between sampling time-points, given that MetS increases over time. For dietary intake data, differences between groups were analyzed by ANCOVA on log-transformed data, after adjustment for energy, sex and percentage of misreporting of energy needs (%EER), to account for known effects on dietary intake, with Bonferroni post-hoc tests. Binary logistic regression was used to identify the macronutrients associated with MetS and its components at follow-up. Drug use was adjusted for as described previously. In addition, sex, study cohort, education, socioeconomic deprivation, energy and percentage of misreporting of energy needs (%EER) were adjusted to account for known effects on dietary intake. For the protein quality data analysis, differences between groups were analyzed by independent samples t-test using log-transformed data. A directed acyclic graph supporting the hypothesized relationships between MetS, diet and the covariates is outlined in Additional file 1: Figure S1. For all analysis, *P* < 0.05 was considered statistically significant.

## Results

### Study participants

**Fig. 1:**
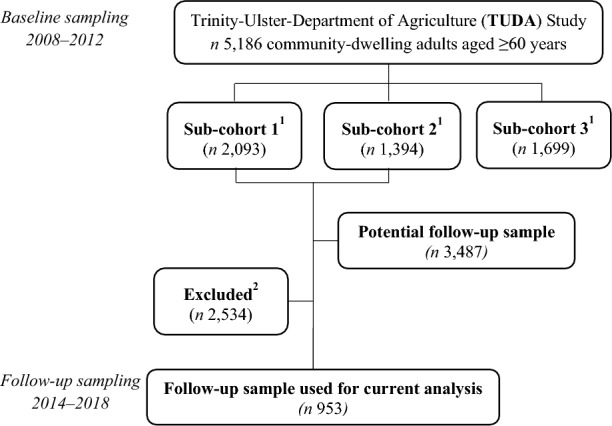
Flow diagram of study design and eligible participants. ^1^Sub-cohort 1 participants had a diagnosis of hypertension and were recruited from General Practice clinics in Northern Ireland. Sub-cohorts 2 and 3 participants were recruited from a specialist bone outpatient service and geriatric outpatient clinics, respectively, at St James Hospital Dublin, Republic of Ireland. Sub-cohort 3 was not included in the follow-up sampling. ^2^Did not meet the study criteria or were unavailable, unable or unwilling for participation in the follow-up study

Identification of the TUDA sample analyzed in this study are outlined in Fig. 1. Of the total 5186 TUDA baseline participants, 3487 were identified as the potential follow-up sample. Participants who were aged < 65 years (*n* 1315) were excluded together with those who had a recorded Folstein Mini-Mental State Examination (MMSE) score < 21 (*n* 39) or were on vitamin B12 injections (*n* 66). A further number of participants (*n* 1114) were uncontactable, unable or unwilling to participate in the follow-up sampling, providing a total of 953 participants who were re-sampled a minimum of 5 years after initial sampling (median follow-up of 5.4 years). Table 1 outlines the general characteristics of the matched TUDA sample at baseline and follow-up (*n* 953). As shown in Table 1, improvements in triglycerides, HDL-and LDL-cholesterol, systolic blood pressure, weight and BMI were observed over time. In contrast, waist circumference, HbA1c concentrations and the proportion of participants who were hyperglycemic or prediabetic increased over time. For comparative purposes, the characteristics at baseline of the total available cohort (*n* 3487) along with the subset who participated in the follow-up study are included in Table 1. As shown in Additional file 1: Table S1, most baseline characteristics of the total available cohort were similar to the baseline characteristics of those who participated in the follow-up study; however, the follow-up participants were generally younger at baseline (*P* < 0.001), were better educated (*P* < 0.001) and lived in areas of higher socioeconomic status (*P* < 0.001).

**Table 1:** General characteristics of the TUDA sample at baseline and follow-up

	Total available cohort	Follow-up sample		
	Baseline (*n* 3487)	Baseline (*n* 953)	Follow-up (*n* 953)	*P*^a^
Age (years)	70.8 (70.6, 71.0)	68.9 (66.6, 71.1)	75.8 (75.5, 76.2)	** < 0.001**
Male sex, *n* (%)	1138 (33)	317 (33)	317 (33)	–
Age formal education ended, *n* (%)				
≤ 14 years	1284 (37)	304 (32)	304 (32)	–
15–16 years	1059 (30)	247 (26)	247 (26)	–
17–18 years	527 (15)	162 (17)	162 (17)	–
≥ 19 years	613 (18)	236 (25)	236 (25)	–
Socioeconomic deprivation, *n* (%)^b^				
Quintile 1	685 (20)	237 (25)	237 (25)	–
Quintile 5	764 (22)	138 (15)	138 (15)	–
MetS^c^ components and related factors				
Waist circumference (cm)	95.7 (95.3, 96.2)	95.2 (94.3, 96.1)	97.7 (96.8, 98.6)	** < 0.001**
Triglycerides (mmol/L)	1.7 (1.6, 1.7)	1.6 (1.6, 1.7)	1.5 (1.5, 1.6)	** < 0.001**
HDL cholesterol (mmol/L)	1.5 (1.5, 1.5)	1.5 (1.5, 1.5)	1.6 (1.6, 1.6)	** < 0.001**
LDL cholesterol (mmol/L)	2.5 (2.5, 2.5)	2.6 (2.5, 2.6)	2.2 (2.2, 2.3)	** < 0.001**
Systolic BP (mmHg)	144.8 (144.1, 145.5)	143.7 (142.4, 144.9)	141.0 (139.7, 142.4)	** < 0.001**
Diastolic BP (mmHg)	79.1 (78.7, 79.4)	78.6 (78.0, 79.3)	80.4 (79.7, 81.1)	** < 0.001**
Hypertensive, *n* (%)^d^	2046 (59)	540 (57)	501 (53)	0.063
HbA1c (mmol/mol)^e^	40.6 (40.3, 40.9)	39.9 (39.4, 40.4)	41.0 (40.2, 41.5)	** < 0.001**
Normoglycemic, *n* (%)	1638 (47)	482 (51)	445 (47)	** < 0.001**
Hyperglycemic, *n* (%)	1687 (48)	432 (45)	494 (52)	** < 0.001**
Prediabetic, *n* (%)	1293 (77)	337 (78)	363 (73)	** < 0.001**
Diabetic, *n* (%)	394 (23)	95 (22)	131 (27)	0.212
Other health and lifestyle factors				
Waist-to-hip ratio (cm)	0.91 (0.91, 0.91)	0.90 (0.90, 0.91)	0.93 (0.92, 0.93)	** < 0.001**
Weight (kg)	74.9 (74.3, 75.4)	75.9 (74.8, 76.9)	74.5 (73.4, 75.6)	** < 0.001**
Height (m)	1.63 (1.63, 1.64)	1.64 (1.63, 1.64)	1.64 (1.64, 1.65)	** < 0.001**
BMI (kg/m^2^)^f^	28.3 (28.2, 28.5)	28.2 (27.9, 28.5)	28.1 (27.7, 28.4)	**0.019**
Overweight, *n* (%)	1392 (40)	389 (41)	385 (40)	0.886
Obese, *n* (%)	1142 (33)	297 (31)	286 (30)	0.334
Timed Up-and-Go (seconds)^g^	10.1 (10.0, 10.3)	9.3 (9.1, 9.6)	11.6 (11.2, 12.0)	** < 0.001**
Physical self-maintenance scale score^h^	23.3 (23.3, 23.4)	23.5 (23.4, 23.6)	23.2 (23.1, 23.3)	** < 0.001**
Physical activity, *n* (%)^i^	2867 (82)	827 (87)	823 (86)	0.746
Living alone, *n* (%)	989 (28)	236 (25)	294 (31)	** < 0.001**
Current smoker, *n* (%)	432 (12)	90 (9)	55 (6)	** < 0.001**
Past smoker, *n* (%)	3055 (88)	385 (40)	422 (44)	** < 0.001**
Alcohol (units/week)^j^	8.4 (7.8, 8.9)	8.0 (7.1, 8.9)	4.5 (4.0, 5.1)	** < 0.001**
Fortified food consumer, *n* (%)^k^	2481 (71)	681 (72)	603 (63)	** < 0.001**
Self-reported medical history				
Diabetes, *n* (%)	424 (12)	95 (10)	135 (14)	** < 0.001**
Hyperlipidemia, *n* (%)	1968 (56)	541 (57)	557 (58)	0.312
Previous myocardial infarction, *n* (%)	309 (9)	77 (8)	90 (9)	**0.031**
Previous TIA, *n* (%)	204 (6)	51 (5)	81 (9)	** < 0.001**
Previous stroke, *n* (%)	105 (3)	16 (2)	25 (3)	0.078

Data expressed as mean (95% CI), except where stated otherwise. This study involved new analysis of existing samples from the Trinity-Ulster-Department of Agriculture (TUDA) cohort (*n* 3487) first sampled in 2008–2012 for comprehensive health, but not dietary, data. The TUDA follow-up sample comprises about 20% of the original cohort who were followed up for re-investigation in 2014–2018 (*n* 953)

BMI, body mass index; HbA1c, hemoglobin A1c; HDL, high-density lipoprotein; LDL, low-density lipoprotein; MetS, metabolic syndrome; TIA, transient ischemic attack

^a^Continuous variables were analyzed using paired samples t-tests on log-transformed data. Categorical variables were analyzed using McNemar’s test.* P* < 0.05 was considered significant; significant values are highlighted in bold text

^b^Area-based socioeconomic deprivation score from individual geo-referenced address-based information, whereby participants were mapped and linked with official socioeconomic indicators of deprivation within Northern Ireland and the Republic of Ireland, as previously described [27]. Deprivation scores were categorized into quintiles (Q1–5), with Q1 being the 20% least deprived category, and Q5 the 20% most deprived category. Q1 and Q5 only shown in Table 1

^c^MetS is a clustering of abnormal metabolic components including abdominal obesity, elevated blood pressure, reduced HDL cholesterol, elevated triglycerides and impaired fasting glucose

^d^Defined as systolic blood pressure (BP) ≥ 140 mmHg and/or diastolic BP ≥ 90 mmHg [74, 75]

^e^HbA1c was used to define participants as normoglycemic (< 39 mmol/mol); hyperglycemic (≥ 39 mmol/mol); prediabetic (≥ 39 to ≤ 47 mmol/mol); and diabetic (≥ 48 mmol/mol) [38]

^f^World Health Organization BMI cut-offs [76]: overweight (≥ 25 to ≤ 29.9 kg/m^2^) and obesity (≥ 30 kg/m^2^). Of note, *n* 58 (2%) of the baseline sample and *n* 16 (2%) of the follow-up sample were identified as underweight (< 18.5 kg/m^2^), while *n* 829 (24%) of the baseline sample and *n* 244 (26%) of the follow-up sample were identified as normal weight (≥ 18.5 to ≤ 24.9 kg/m^2^)

^g^Timed Up-and-Go test measured the time taken to stand up from seated in a chair, walk three meters, turn around and walk back to return to the original seated position

^h^The physical self-maintenance scale questionnaire assigns scores to the participants highest level of functioning for activities of daily living, the higher the total score the more independent the participant

^i^Any exercise in the last two weeks

^j^Alcohol units per week among those consuming alcohol: *n* 2167 (62%) of the baseline sample; *n* 602 (63%) of the follow-up sample. One unit equates with 25 mL spirits, 220 mL beer, and 85 mL wine

^k^Participants who consumed foods fortified with B-vitamins at least once per week

### Proportion of participants affected by MetS and its components

The proportions of participants from the follow-up investigation who were affected by MetS and its components at baseline and follow-up are outlined in Table 2. The prevalence of MetS is shown to significantly increase over time (67% at baseline *vs*. 74% at follow-up; *P* < 0.001). The proportions of participants affected by each MetS component also increased with advancing age, except for triglycerides and HDL-cholesterol where improvements were observed with advancing age. Of note, a small proportion of participants (*n* 76) had MetS at baseline but no longer had it at follow-up (the baseline and follow-up characteristics of these *n* 76 participants are outlined in Additional file 1: Table S2).

**Table 2:** Proportions of male and female participants affected by the metabolic syndrome (MetS)^a^ and its components at baseline and follow-up

	Baseline			Follow-up			
	Total (*n* 953)	Males (*n* 317)	Females (*n* 636)	Total (*n* 953)	Males (*n* 317)	Females (*n* 636)	*P*^b^
Metabolic syndrome, *n (%)*	637 (67)	242 (76)	395 (62)	705 (74)	260 (82)	445 (70)	** < 0.001**
Abdominal obesity, *n (%)*	519 (55)	175 (55)	344 (54)	606 (64)	197 (62)	409 (64)	** < 0.001**
Hypertension, *n (%)*	844 (89)	310 (98)	534 (84)	870 (91)	310 (98)	560 (88)	** < 0.001**
Hyperglycemia, *n (%)*	438 (46)	163 (51)	275 (43)	496 (52)	188 (59)	308 (49)	** < 0.001**
Dyslipidemia, *n (%)*	686 (72)	261 (82)	425 (67)	706 (74)	262 (83)	444 (70)	** < 0.001**
Raised triglycerides, *n (%)*	349 (37)	154 (49)	195 (31)	290 (30)	111 (35)	179 (28)	** < 0.001**
Reduced HDL-c, *n (%)*	227 (24)	83 (26)	144 (23)	149 (16)	61 (19)	88 (14)	** < 0.001**

Data expressed as* n* (%). Data obtained from the Trinity-Ulster-Department of Agriculture (TUDA) baseline sample (2008–2012; *n* 953) and the corresponding follow-up sample (2014–2018; *n* 953)

*HDL-c* high-density lipoprotein cholesterol

^a^Participants were deemed to have MetS if they met at least three of the following criteria: waist circumference of ≥ 102 cm or ≥ 88 cm, for males and females respectively [37]; elevated triglycerides of ≥ 1.7 mmol/L (≥ 150 mg/dL) [4]; reduced HDL cholesterol of < 1.0 mmol/L (< 40 mg/dL) for males and < 1.3 mmol/L (< 50 mg/dL) for females [4]; elevated blood pressure of systolic ≥ 130 and/or diastolic ≥ 85 mmHg [4]; and HbA1c of ≥ 39 mmol/mol [38]

^b^Differences between the total sample at baseline and at follow-up were analyzed by chi-square; *P* < 0.05 was considered significant; significant values are highlighted in bold text

### Baseline factors associated with higher MetS risk and its progression over time

Binary logistic regression was used to identify baseline factors associated with higher MetS risk and its progression over time (Table 3). After adjustment for anti-hypertensive, diabetic and lipid-lowering drug use, waist circumference and triglycerides were found to be significant predictors of a higher MetS risk at follow-up. When predictors of each component of MetS were examined individually, living in the most deprived socioeconomic areas, waist circumference and BMI were found to be significant predictors of abdominal obesity risk at follow-up, whereas male sex and HbA1c concentrations predicted a lower risk. After adjustment for anti-hypertensive drug use, alcohol intake, HDL cholesterol and systolic BP were found to be predictors of hypertension risk at follow-up. When adjusted for diabetic drug use, HbA1c was found to be a predictor of hyperglycemia risk at follow-up, while being in sub-cohort 2 (the bone cohort) predicted a lower risk. Triglycerides were found to be a predictor of dyslipidemia risk at follow-up, while HDL cholesterol predicted a lower risk, after adjustment for lipid-lowering drug use.

**Table 3:** Predictors at baseline for the development of metabolic syndrome (MetS)^a^ and its components at follow-up

	MetS		Components of MetS							
			Abdominal obesity^b^		Hypertension^c^		Hyperglycemia^d^		Dyslipidemia^e^	
	Odds Ratio (95% CI) (*n* 705 *vs* 248)	*P*	Odds Ratio (95% CI) (*n* 606 *vs* 339)	*P*	Odds Ratio (95% CI) (*n* 870 *vs* 83)	*P*	Odds Ratio (95% CI) (*n* 496 *vs* 457)	*P*	Odds Ratio (95% CI) (*n* 706 *vs* 247)	*P*
General factors										
Age, years	0.98 (0.92, 1.04)	0.467	1.02 (0.96, 1.09)	0.496	0.98 (0.85, 1.12)	0.738	1.00 (0.95, 1.05)	0.862	1.00 (0.92, 1.08)	0.992
Male sex	0.46 (0.20, 1.08)	0.075	0.06 (0.02, 0.15)	** < 0.001**	1.28 (0.09, 6.95)	0.860	0.84 (0.44, 1.60)	0.591	0.83 (0.32, 2.16)	0.709
Study cohort^f^	1.11 (0.23, 5.50)	0.894	1.60 (0.34, 7.52)	0.550	0.00 (0.00, 0.00)	0.990	0.37 (0.20, 0.68)	**0.001**	1.03 (0.15, 7.18)	0.976
Education, years^g^	1.03 (0.95, 1.12)	0.523	0.98 (0.90, 1.06)	0.589	1.09 (0.93, 1.27)	0.301	1.02 (0.96, 1.09)	0.461	1.08 (0.97, 1.21)	0.161
Socioeconomic deprivation^h^	1.01 (0.53, 1.91)	0.987	1.98 (1.07, 3.68)	**0.031**	1.64 (0.51, 5.25)	0.405	1.01 (0.61, 1.67)	0.970	1.23 (0.57, 2.67)	0.595
TUG, s^i^	1.04 (0.92, 1.18)	0.546	1.05 (0.93, 1.20)	0.417	1.08 (0.86, 1.36)	0.510	1.03 (0.95, 1.12)	0.424	0.97 (0.83, 1.12)	0.646
PSMS score^j^	1.14 (0.82, 1.57)	0.436	1.02 (0.74, 1.40)	0.913	0.73 (0.38, 1.38)	0.332	1.02 (0.79, 1.32)	0.872	1.10 (0.75, 1.62)	0.628
Exercise, *n* (%)^k^	1.20 (0.42, 3.36)	0.739	0.91 (0.35, 2.36)	0.848	7.02 (0.56, 88.67)	0.186	1.32 (0.61, 2.85)	0.486	1.77 (0.38, 8.15)	0.466
Living alone, *n* (%)	1.43 (0.74, 2.80)	0.291	0.90 (0.47, 1.70)	0.736	0.32 (0.09, 1.08)	0.066	1.09 (0.66, 1.82)	0.737	1.72 (0.76, 3.89)	0.193
Current smoker, *n* (%)	0.59 (0.23, 1.52)	0.271	0.84 (0.33, 2.15)	0.716	1.35 (0.28, 6.52)	0.712	0.61 (0.28, 1.33)	0.214	1.84 (0.62, 5.44)	0.269
Past smoker, *n* (%)	0.78 (0.48, 1.28)	0.320	1.21 (0.68, 2.15)	0.514	0.43 (0.12, 1.57)	0.201	0.88 (0.56, 1.40)	0.590	0.60 (0.28, 1.28)	0.184
Alcohol (units/week)^l^	0.98 (0.96, 1.00)	0.091	0.98 (0.96, 1.01)	0.165	1.18 (1.02, 1.37)	**0.023**	1.00 (0.98, 1.01)	0.578	1.00 (0.98, 1.03)	0.754
MetS factors										
Waist (cm)	1.06 (1.01, 1.11)	**0.016**	1.16 (1.11, 1.22)	** < 0.001**	0.97 (0.89, 1.05)	0.434	1.00 (0.97, 1.03)	0.923	1.01 (0.97, 1.05)	0.613
BMI (kg/m^2^)	0.98 (0.87, 1.10)	0.718	1.43 (1.24, 1.64)	** < 0.001**	1.10 (0.88, 1.37)	0.411	0.99 (0.90, 1.08)	0.776	0.96 (0.86, 1.09)	0.545
TG (mmol/L)	2.01 (1.29, 3.16)	**0.002**	1.20 (0.81, 1.80)	0.363	1.58 (0.52, 4.81)	0.418	0.97 (0.70, 1.35)	0.861	3.49 (1.96, 6.22)	** < 0.001**
HDL-c (mmol/L)	0.78 (0.43, 1.41)	0.405	1.06 (0.54, 2.07)	0.875	4.49 (1.10, 18.28)	**0.036**	0.75 (0.41, 1.36)	0.344	0.29 (0.12, 0.71)	**0.006**
LDL-c (mmol/L)	0.93 (0.67, 1.29)	0.654	0.86 (0.64, 1.15)	0.303	1.04 (0.58, 1.88)	0.889	0.94 (0.73, 1.19)	0.593	0.76 (0.51, 1.13)	0.173
Systolic BP (mmHg)	1.00 (0.99, 1.02)	0.723	0.98 (0.97, 1.00)	0.057	1.11 (1.04, 1.18)	**0.002**	0.99 (0.98, 1.00)	0.134	1.02 (1.00, 1.05)	0.073
Diastolic BP (mmHg)	1.00 (0.97, 1.04)	0.876	1.00 (0.97, 1.03)	0.862	1.00 (0.91, 1.11)	0.944	1.00 (0.98, 1.04)	0.550	0.99 (0.94, 1.04)	0.604
HbA1c (mmol/mol)	1.03 (0.97, 1.11)	0.315	0.96 (0.92, 1.00)	**0.042**	1.04 (0.90, 1.21)	0.567	1.48 (1.35, 1.61)	** < 0.001**	1.04 (0.98, 1.11)	0.212

Values shown are odds ratio (95% CI). Predictor data obtained from the Trinity-Ulster-Department of Agriculture (TUDA) baseline sample (*n* 953); outcome data obtained from the corresponding follow-up sample (*n* 953). Binary logistic regression analysis adjusting for anti-hypertensive drugs, diabetic drugs and lipid-lowering drugs where relevant, and the time interval between sampling (months), reference category is without the outcome variable. *P* < 0.05 was considered significant; significant values are highlighted in bold text

*BMI* body mass index, *HbA1c* hemoglobin *A1c* HDL-c high-density lipoprotein cholesterol, *LDL-c* low-density lipoprotein cholesterol, *TG* triglycerides

^a^MetS is a clustering of abnormal metabolic components including abdominal obesity, elevated blood pressure, reduced HDL cholesterol, elevated triglycerides and impaired fasting glucose

^b^Defined as waist circumference of ≥ 102 cm or ≥ 88 cm, for males and females respectively

^c^Defined as systolic blood pressure (BP) ≥ 130 and/or diastolic BP ≥ 85 mmHg or on an anti-hypertensive drug

^d^Defined as HbA1c ≥ 39 mmol/mol or on a diabetic drug

^e^Defined as elevated triglycerides (≥ 1.7 mmol/L) or reduced HDL cholesterol (< 1.0 mmol/L for males; < 1.3 mmol/L for females) or on a statin or other lipid-lowering drug

^f^Comparing the bone sub-cohort to the hypertensive sub-cohort (reference category)

^g^Age formal education ended

^h^Area-based socioeconomic deprivation score from individual geo-referenced address-based information, as previously described [27]. Deprivation scores were categorized into quintiles (Q1–5), with Q1 being the 20% least deprived category, and Q5 the 20% most deprived category. For this analysis, participants in Q1, Q2 and Q3 were grouped into ‘less deprived’ and participants in Q4 and Q5 were grouped into ‘more deprived’. The reference category is less deprived

^i^Timed Up-and-Go (TUG) test measured the time taken to stand up from seated in a chair, walk three meters, turn around and walk back to return to the original seated position

^j^The physical self-maintenance scale (PSMS) questionnaire assigns scores to the participants highest level of functioning for activities of daily living, the higher the total score the more independent the participant

^k^Any exercise in the last 2 weeks

^l^Alcohol units per week among those consuming alcohol. One unit equates with 25 mL spirits, 220 mL beer, and 85 mL wine

### Progression of nutrition-related factors and MetS characteristics over time

The progression of nutrition-related factors and MetS characteristics over time were examined and are outlined in Table 4. In participants with MetS at baseline, anti-hypertensive and diabetic medication usage increased over time. Improvements in triglycerides, HDL-cholesterol, LDL-cholesterol, systolic blood pressure and weight were observed over time. In contrast, waist circumference, HbA1c concentrations and the percentage who were diabetic increased over time. Similar observations were noted in participants who did not have MetS at baseline. Lipid-lowering and anti-hypertensive medication usage increased over time. While HDL-cholesterol, LDL-cholesterol and weight improved over time, waist circumference, diastolic blood pressure, HbA1c concentrations and the percentage who were prediabetic increased over time. In addition, a higher proportion of participants with, compared to those without, MetS were male (38% *vs*. 24%), lived in the most deprived areas (31% *vs*. 28%) and finished formal education at a younger age (16.6 years *vs*. 17.6 years). Furthermore, a higher proportion of participants with MetS were taking lipid-lowering, anti-hypertensive and diabetic medications, than those without MetS. Additional file 1: Table S3 provides details on the nutrition-related factors and MetS characteristics of males and females with and without MetS at follow-up only.

**Table 4:** Progression of nutrition-related factors and metabolic syndrome (MetS)^a^ characteristics over time in TUDA participants

	With MetS at baseline (*n* 637)			Without MetS at baseline (*n* 316)		
	Baseline	Follow-up	*P* value	Baseline	Follow-up	*P* value
Age (years)	69.1 (65.8, 72.5)	76.3 (75.9, 76.7)	** < 0.001**	68.3 (67.7, 68.9)	74.8 (74.3, 75.3)	** < 0.001**
Male sex, *n* (%)	242 (38)	242 (38)	–	75 (24)	75 (24)	–
Age formal education ended (years)	16.6 (16.3, 16.8)	16.6 (16.3, 16.8)	–	17.6 (17.2, 18.0)	17.6 (17.2, 18.0)	–
Socioeconomic deprivation, *n* (%)^b^						
Less deprived (Q1, Q2, Q3)	413 (65)	413 (65)	–	220 (70)	220 (70)	–
More deprived (Q4, Q5)	200 (31)	200 (31)	–	88 (28)	88 (28)	–
Drug treatments						
Lipid-lowering drugs*, n* (%)	471 (74)	473 (74)	0.914	39 (12)	107 (34)	** < 0.001**
Anti-hypertensive drugs, *n* (%)	532 (84)	559 (88)	**0.001**	150 (48)	189 (60)	** < 0.001**
Diabetic drugs*, n* (%)	78 (12)	100 (16)	** < 0.001**	2 (1)	5 (2)	0.250
MetS components and related factors						
Waist circumference (cm)	99.6 (98.6, 100.7)	101.6 (100.5, 102.6)	** < 0.001**	86.3 (85.0, 87.6)	89.8 (88.5, 91.2)	** < 0.001**
Triglycerides (mmol/L)	1.8 (1.7, 1.9)	1.7 (1.6, 1.7)	** < 0.001**	1.3 (1.3, 1.4)	1.3 (1.2, 1.4)	0.854
HDL cholesterol (mmol/L)	1.4 (1.4, 1.4)	1.5 (1.4, 1.5)	** < 0.001**	1.7 (1.7, 1.8)	1.9 (1.8, 1.9)	** < 0.001**
LDL cholesterol (mmol/L)	2.3 (2.3, 2.4)	2.1 (2.0, 2.2)	** < 0.001**	3.0 (2.9, 3.1)	2.5 (2.4, 2.6)	** < 0.001**
Systolic BP (mmHg)	146.5 (144.9, 148.0)	142.6 (140.9, 144.2)	** < 0.001**	137.9 (135.7, 140.2)	137.9 (135.6, 140.2)	0.844
Diastolic BP (mmHg)	78.8 (78.0, 79.6)	79.5 (78.6, 80.3)	0.194	77.4 (77.3, 79.6)	82.3 (81.0, 83.5)	** < 0.001**
Hypertensive, *n* (%)^c^	402 (63)	355 (56)	**0.002**	138 (44)	146 (46)	0.303
HbA1c (mmol/mol)^d^	41.6 (40.9, 42.4)	42.8 (42.0, 43.5)	** < 0.001**	36.2 (35.8, 36.7)	37.3 (36.8, 37.8)	** < 0.001**
Normoglycemic, *n* (%)	231 (36)	232 (36)	0.533	251 (79)	213 (67)	** < 0.001**
Hyperglycemic, *n* (%)	391 (61)	400 (63)	0.533	41 (13)	94 (30)	** < 0.001**
Prediabetic, *n* (%)	298 (76)	276 (69)	0.087	39 (95)	87 (93)	** < 0.001**
Diabetic, *n* (%)	93 (24)	124 (31)	** < 0.001**	2 (5)	7 (7)	0.375
Other health and lifestyle factors						
Waist-to-hip ratio (cm)	0.92 (0.92, 0.93)	0.94 (0.93, 0.95)	** < 0.001**	0.86 (0.85, 0.87)	0.90 (0.89, 0.90)	** < 0.001**
Weight (kg)	80.3 (79.0, 81.7)	78.7 (77.4, 80.1)	** < 0.001**	66.9 (65.5, 68.3)	65.9 (64.4, 67.4)	** < 0.001**
Height (m)	1.7 (1.6, 1.7)	1.6 (1.6, 1.6)	** < 0.001**	1.6 (1.6, 1.6)	1.6 (1.6, 1.6)	** < 0.001**
BMI (kg/m^2^)^e^	29.6 (29.3, 30.0)	29.4 (29.0, 29.8)	**0.008**	25.4 (24.9, 25.8)	25.4 (24.9, 25.8)	0.711
Overweight, *n* (%)	271 (43)	274 (43)	0.800	118 (37)	111 (35)	0.418
Obese, *n* (%)	260 (41)	245 (39)	0.110	37 (12)	41 (13)	0.359
Timed Up-and-Go (seconds)^f^	9.6 (9.4, 9.8)	11.9 (11.4, 12.4)	** < 0.001**	8.8 (8.3, 9.3)	11.1 (10.6, 11.6)	** < 0.001**
Physical self-maintenance scale score^g^	23.4 (23.3, 23.5)	23.1 (22.9, 23.2)	** < 0.001**	23.7 (23.6, 23.8)	23.5 (23.4, 23.6)	**0.003**
Physical activity, *n* (%)^h^	531 (83)	523 (82)	0.471	296 (94)	300 (95)	0.584
Living alone, *n* (%)	154 (24)	194 (31)	** < 0.001**	82 (26)	100 (32)	** < 0.001**
Current smoker, *n* (%)	55 (9)	38 (6)	** < 0.001**	35 (11)	17 (5)	** < 0.001**
Past smoker, *n* (%)	275 (43)	294 (46)	**0.017**	110 (35)	128 (41)	**0.009**
Alcohol (units/week)^i^	8.3 (7.1, 9.6)	4.5 (3.9, 5.2)	**0.021**	7.4 (6.2, 8.7)	4.6 (3.7, 5.4)	** < 0.001**
Fortified food consumer, *n* (%)^j^	453 (71)	402 (63)	** < 0.001**	228 (72)	201 (64)	**0.010**
Self-reported medical history						
Diabetes, *n* (%)	94 (15)	128 (20)	** < 0.001**	1 (1)	7 (2)	**0.031**
Hyperlipidemia, *n* (%)	454 (71)	425 (67)	**0.017**	87 (28)	132 (42)	** < 0.001**
Previous myocardial infarction, *n* (%)	69 (11)	79 (12)	**0.002**	8 (3)	11 (4)	0.250
Previous transient ischemic attack, *n* (%)	46 (7)	67 (11)	0.089	5 (2)	14 (4)	**0.022**
Previous stroke, *n* (%)	14 (2)	21 (3)	0.167	2 (1)	4 (1)	0.500

Data expressed as mean (95% CI), except where stated otherwise. Data obtained from the Trinity-Ulster-Department of Agriculture (TUDA) baseline sample (2008–2012) and the corresponding follow-up sample (2014–2018; *n* 953). Continuous variables were analyzed using paired samples t-tests on log-transformed data. Categorical variables were analyzed using McNemar’s test.* P* < 0.05 was considered significant; significant values are highlighted in bold text

*BMI* body mass index, *HbA1c* hemoglobin A1c; *HDL* high-density lipoprotein, *LDL* low-density lipoprotein

^a^Participants were deemed to have MetS if they met at least three of the following criteria: waist circumference of ≥ 102 cm or ≥ 88 cm, for males and females respectively [37]; elevated triglycerides of ≥ 1.7 mmol/L (≥ 150 mg/dL) [4]; reduced HDL cholesterol of < 1.0 mmol/L (< 40 mg/dL) for males and < 1.3 mmol/L (< 50 mg/dL) for females [4]; elevated blood pressure of systolic ≥ 130 and/or diastolic ≥ 85 mmHg [4]; and HbA1c of ≥ 39 mmol/mol [38]

^b^Area-based socioeconomic deprivation score from individual geo-referenced address-based information, as previously described [27]. Deprivation scores were categorized into quintiles (Q1–5), with Q1 being the 20% least deprived category, and Q5 the 20% most deprived category. For this analysis, participants in Q1, Q2 and Q3 were grouped into ‘less deprived’ and participants in Q4 and Q5 were grouped into ‘more deprived’

^c^Defined as systolic blood pressure (BP) ≥ 140 mmHg and/or diastolic BP ≥ 90 mmHg [74, 75]

^d^HbA1c was used to define participants as normoglycemic (< 39 mmol/mol); hyperglycemic (≥ 39 mmol/mol); prediabetic (≥ 39 to ≤ 47 mmol/mol); and diabetic (≥ 48 mmol/mol) [38]

^e^World Health Organization BMI cut-offs [76] were used to define overweight (≥ 25 to ≤ 29.9 kg/m^2^) and obesity (≥ 30 kg/m^2^)

^f^Timed Up-and-Go test measured the time taken to stand up from seated in a chair, walk three meters, turn around and walk back to return to the original seated position

^h^The physical self-maintenance scale is a questionnaire which assigns scores to the participants highest level of functioning for activities of daily living, the higher the total score the more independent the participant

^i^Any exercise in the last 2 weeks

^j^Alcohol units per week among those consuming alcohol: *n* 345 (62%) of the participants with MetS at baseline sampled at baseline, *n* 320 (57%) of the participants with MetS at baseline sampled at follow-up; *n* 234 (74%) of the participants without MetS at baseline sampled at baseline, *n* 228 (72%) of the participants without MetS at baseline sampled at follow-up. One unit equates with 25 mL spirits, 220 mL beer, and 85 mL wine

^k^Participants who consumed foods fortified with B-vitamins at least once per week

### Daily energy and macronutrient intakes of participants with and without MetS at follow-up

The daily energy and macronutrient intakes of participants with and without MetS are presented in Table 5. Of the 953 follow-up participants, corresponding dietary intake data was available for *n* 803 (84%). Participants with MetS had significantly lower intakes of energy, protein, polyunsaturated fatty acids (PUFA) and fiber. Participants with MetS also had significantly higher intakes of carbohydrate, starch and free sugar. While potential mis-reporters were not excluded from the analysis, it is worth noting that 23% of participants with MetS and 13% of participants without MetS were identified as potential mis-reporters. Additional file 1: Table S4 provides the daily energy and macronutrient intakes of participants with and without MetS, split by sex. Additional file 1: Table S5 outlines the food groups contributing to protein intake in participants with and without MetS, split by sex.

**Table 5:** Daily energy and macronutrient intakes of Irish older adults with and without metabolic syndrome (MetS)^a^

	With MetS (*n* 596)	Without MetS (*n* 207)	*P* value	DRV^b^
Energy (MJ)	7.16 (2.34)	7.29 (2.23)	** < 0.001**	8.4–11.9 (males) 6.8–9.6 (females)
Energy (kcal)^c^	1708 (557)	1734 (533)	** < 0.001**	2017–2834 (males) 1628–2305 (females)
Protein (g)	74.0 (23.2)	76.6 (22.0)	**0.005**	–
Protein (%EI)	16.9 (4.1)	17.3 (4.2)	** < 0.001**	–
Protein (g/kg bw)	0.96 (0.40)	1.19 (0.40)	** < 0.001**	0.83
Total Fat (g)	64.7 (28.5)	70.0 (26.4)	0.341	–
Total Fat (%EI)	34.9 (7.0)	35.1 (6.8)	0.286	20–35
Saturated fat (g)	25.6 (13.0)	25.2 (13.5)	0.228	–
Saturated Fat (%EI)	13.5 (4.5)	13.2 (4.3)	0.234	≤ 10%EI^d^
MUFA (g)	22.1 (10.1)	23.8 (10.2)	0.096	–
MUFA (%EI)	11.7 (3.0)	12.0 (3.0)	0.087	–
PUFA (g)	9.0 (5.0)	10.0 (5.1)	0.051	–
PUFA (%EI)	4.7 (2.1)	5.0 (2.4)	**0.022**	–
DHA + EPA (mg)	34.1 (33.0)	38.6 (42.0)	0.193	250
Carbohydrate (g)	198.4 (75.6)	188.7 (74.2)	** < 0.001**	–
Carbohydrate (%EI)	47.1 (7.5)	44.7 (8.9)	** < 0.001**	45–60
Starch (g)	102.7 (45.9)	96.2 (47.2)	** < 0.001**	–
Total Sugar (g)	84.2 (40.4)	85.4 (43.9)	0.449	–
Free Sugar (g)	31.8 (28.7)	30.8 (32.6)	**0.040**	ALAP
Free Sugar (%EI)	7.8 (5.9)	7.2 (6.0)	**0.020**	< 10%EI^e^ < 5%EI^e^
Fiber (g)	18.7 (7.7)	20.1 (8.0)	**0.049**	25

Data expressed as median (IQR). Data obtained from the Trinity-Ulster-Department of Agriculture (TUDA) follow-up sample where dietary data was available for *n* 803. Variables were analyzed by ANCOVA (adjusting for energy, sex and percentage of misreporting of energy needs (%EER)) on log-transformed data as appropriate with Bonferroni post-hoc tests. *P* < 0.05 was considered significant; significant values are highlighted in bold text

*%EI* % energy intake, *ALAP* as low as possible; bw, body weight, *DHA* docosahexaenoic acid, *EPA* eicosapentaenoic acid, *HbA1c* hemoglobin A1c, *MUFA* monounsaturated fatty acid, *PUFA* polyunsaturated fatty acid

^a^Participants were deemed to have MetS if they met at least three of the following criteria: waist circumference of ≥ 102 cm or ≥ 88 cm, for males and females respectively [37]; elevated triglycerides of ≥ 1.7 mmol/L (≥ 150 mg/dL) [4]; reduced HDL cholesterol of < 1.0 mmol/L (< 40 mg/dL) for males and < 1.3 mmol/L (< 50 mg/dL) for females [4]; elevated blood pressure of systolic ≥ 130 and/or diastolic ≥ 85 mmHg [4]; and HbA1c of ≥ 39 mmol/mol [38]

^b^European Food Safety Authority (EFSA) Dietary Reference Values (DRVs) for energy and each macronutrient, where applicable [71]

^c^Of note, 23% of participants with MetS and 13% of participants without MetS were identified as potential mis-reporters. Potential misreporting was estimated using predicted values for basal metabolic rate (Oxford equations) [31] and physical activity levels [32]. Potential mis-reporters were not excluded from analysis

^d^World Health Organization strong recommendation [69]

^e^Free sugar limits of < 10% energy intake and < 5% energy intake were obtained from World Health Organization guidelines [70]

### Associations of macronutrients with MetS and its components at follow-up

Binary logistic regression was used to identify dietary determinants of MetS and its components at follow-up (Table 6). Higher protein (g/kg bw/day) and monounsaturated fatty acid (g/day) intakes were each associated with lower risk of MetS, whilst higher protein (g/kg bw/day) intake was also associated with lower abdominal obesity and hypertension.

**Table 6:** Associations of macronutrients with the metabolic syndrome (MetS)^a^ and its components at follow-up

	MetS		Components of MetS							
			Abdominal obesity^b^		Hypertension^c^		Hyperglycemia^d^		Dyslipidemia^e^	
	Odds Ratio (95% CI) (*n* 596 *vs* 207)	*P*	Odds Ratio (95% CI) (*n* 502 *vs* 295)	*P*	Odds Ratio (95% CI) (*n* 733 *vs* 70)	*P*	Odds Ratio (95% CI) (*n* 419 *vs* 381)	*P*	Odds Ratio (95% CI) (*n* 600 *vs* 202)	*P*
Age (y)	0.98 (0.92, 1.04)	0.524	1.03 (0.98, 1.09)	0.195	1.14 (1.02, 1.28)	**0.023**	1.01 (0.97, 1.05)	0.555	0.97 (0.91, 1.04)	0.403
Energy (kcal)	0.96 (0.90, 1.04)	0.329	0.93 (0.85, 1.02)	0.127	1.02 (0.76, 1.36)	0.909	1.01 (0.95, 1.07)	0.696	0.81 (0.55, 1.21)	0.308
Protein (g/kg bw)	0.06 (0.02, 0.20)	** < 0.001**	0.10 (0.02, 0.51)	**0.006**	0.22 (0.00, 0.80)	**0.037**	0.62 (0.17, 2.26)	0.466	0.22 (0.01, 5.93)	0.368
Total fat (g)	1.05 (0.96, 1.15)	0.290	1.04 (0.96, 1.14)	0.324	0.91 (0.81, 1.03)	0.134	1.01 (0.95, 1.08)	0.737	0.97 (0.86, 1.09)	0.593
Saturated fat (g)	1.00 (0.96, 1.10)	0.924	0.98 (0.89, 1.08)	0.694	1.07 (0.93, 1.24)	0.334	1.02 (0.95, 1.09)	0.633	1.09 (0.97, 1.23)	0.143
MUFA (g)	0.88 (0.78, 1.00)	**0.030**	0.94 (0.84, 1.04)	0.232	0.92 (0.78, 1.08)	0.314	0.97 (0.89, 1.05)	0.405	0.99 (0.86, 1.13)	0.862
PUFA (g)	0.97 (0.86, 1.09)	0.563	0.98 (0.88, 1.10)	0.746	1.19 (1.00, 1.42)	0.065	0.92 (0.84, 1.00)	0.062	0.98 (0.85, 1.13)	0.759
DHA + EPA (mg)	1.00 (0.99, 1.00)	0.271	1.00 (0.99, 1.01)	0.724	1.01 (1.00, 1.03)	0.190	1.00 (0.99, 1.00)	0.554	0.99 (0.99, 1.00)	0.242
Carbohydrate (g)	1.01 (0.98, 1.04)	0.675	1.00 (0.97, 1.03)	0.893	0.99 (0.93, 1.06)	0.780	1.01 (0.99, 1.04)	0.235	1.00 (0.95, 1.05)	0.979
Starch (g)	1.00 (0.98, 1.03)	0.972	0.99 (0.96, 1.01)	0.281	1.04 (0.97, 1.10)	0.258	0.99 (0.97, 1.01)	0.422	0.99 (0.96, 1.02)	0.559
Total Sugar (g)	0.98 (0.95, 1.01)	0.256	0.98 (0.96, 1.01)	0.251	0.98 (0.92, 1.05)	0.593	0.99 (0.97, 1.01)	0.289	0.97 (0.94, 1.01)	0.171
Free Sugar (g)	1.01 (0.99, 1.03)	0.422	0.99 (0.98, 1.01)	0.472	1.02 (0.99, 1.06)	0.237	1.00 (0.99, 1.01)	0.741	1.02 (0.99, 1.04)	0.191
Fiber (g)	0.99 (0.92, 1.06)	0.677	1.01 (0.96, 1.07)	0.626	0.92 (0.81, 1.03)	0.148	0.92 (0.95, 1.04)	0.747	0.98 (0.90, 1.07)	0.615

Values shown are odds ratio (95% CI). Data obtained from the Trinity-Ulster-Department of Agriculture (TUDA) follow-up sample where dietary data was available for *n* 803. Binary logistic regression analysis (adjusting for sex, study cohort, education, socioeconomic deprivation, percentage of misreporting of energy needs (%EER), energy (MJ) and anti-hypertensive drugs, diabetic drugs and lipid-lowering drugs where relevant), reference category is without the outcome variable. *P* < 0.05 was considered significant; significant values are highlighted in bold text

*bw* body weight, *DHA* docosahexaenoic acid, *EPA* eicosapentaenoic acid, *HbA1c* hemoglobin A1c; *HDL* high-density lipoprotein, *MUFA*, monounsaturated fatty acid, *PUFA* polyunsaturated fatty acid

^a^MetS is a clustering of abnormal metabolic components including abdominal obesity, elevated blood pressure, reduced HDL cholesterol, elevated triglycerides and impaired fasting glucose

^b^Abdominal obesity is defined as waist circumference of ≥ 102 cm or ≥ 88 cm, for males and females respectively

^c^Hypertension is defined as systolic blood pressure (BP) ≥ 130 and/or diastolic BP ≥ 85 mmHg

^d^Hyperglycemia is defined as HbA1c ≥ 39 mmol/mol

^e^Dyslipidemia is defined as elevated triglycerides (≥ 1.7 mmol/L) or reduced HDL cholesterol (< 1.0 mmol/L for males; < 1.3 mmol/L for females)

### Protein quality of foods consumed by participants with and without MetS

**Fig. 2:**
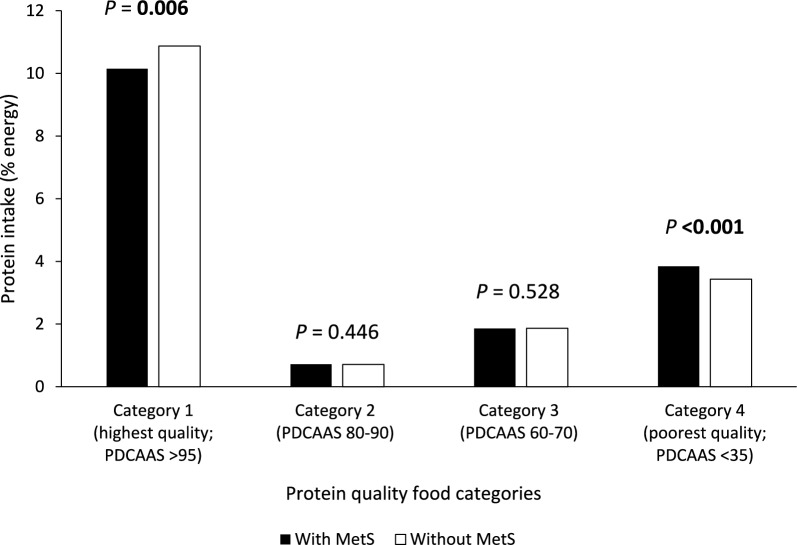
Protein intake (% energy intake) from the four protein quality food categories^1^ in participants with and without metabolic syndrome (MetS)^2^ at follow-up. Dietary data from the Trinity-Ulster-Department of Agriculture (TUDA) follow-up sample, available for *n* 803. Differences between groups were analyzed by independent samples t-test on log-transformed data; *P* < 0.05 was considered significant; significant values are highlighted in bold text. ^1^Protein quality was assessed using the protein digestibility-corrected amino acid score (PDCAAS). The higher the PDCAAS, the better the quality of the protein. The protein quality categories were defined as follows: category 1 (PDCAAS > 95), category 2 (PDCASS 80–90), category 3 (PDCAAS 60–70) and category 4 (PDCAAS < 35). ^2^MetS is a clustering of abnormal metabolic components including abdominal obesity, elevated blood pressure, reduced HDL cholesterol, elevated triglycerides and impaired fasting glucose. *HDL* high-density lipoprotein

Protein intake (as %EI) from each of the four protein quality food categories in participants with and without MetS are outlined in Fig. 2. In participants with MetS, significantly less protein (%EI) was consumed as high-quality protein foods (category 1, PDCAAS > 95) compared to participants without MetS (10%EI *vs*. 11%EI, respectively; *P* = 0.006), while significantly more protein (%EI) was consumed as low-quality protein foods (category 4, PDCAAS < 35; 4%EI *vs*. 3%EI, respectively, *P* < 0.001). High-quality protein foods included meat, dairy and soy products, while low-quality protein foods mostly included breads and confectionary products. There were no significant differences in the quality of protein foods consumed by the least deprived and most deprived socioeconomic status groups (Additional file 1: Figure S2).

## Discussion

We investigated nutrition factors in relation to MetS and its progression over a follow-up period of 5.4 years in older adults. Predictors at baseline for the development of MetS at follow-up were higher waist circumference (but not BMI) and increased triglyceride concentrations. Higher dietary intakes of protein and MUFA were associated with a lower risk of MetS. Participants with MetS, compared to those without, had lower protein and fiber intakes, and notably consumed less high-quality and more low-quality protein foods.

Using a recent harmonized definition [4], MetS affected 67% and 74% of participants, at baseline and follow-up respectively. The use of various MetS definitions makes it difficult to compare studies; however the high prevalence of MetS in the current study broadly aligns with rates reported in other studies of older adults using this definition [39–41], whereas studies using alternative MetS definitions generally report lower rates [42], with one recent study of Irish adults (≥ 50 years) reporting a prevalence of just 40% using the IDF and ATPIII definitions [21]. Also of note, a small proportion (12%) of participants who had MetS at baseline in the current study no longer had MetS at follow-up. These participants had improved lipid profiles, blood pressure, blood glucose, and BMI at follow-up, most likely due to improvements in diet, lifestyle and medical interventions. This finding supports the potential to reverse MetS and its components through effective strategies targeting risk factors [9, 21]. Abdominal obesity has been reported as the most prevalent MetS component [40, 43], however in this study hypertension was more prevalent, possibly related to the recruitment of participants on the basis of having a diagnosis of hypertension (62%).

Consistent with previous reports, we observed an overall higher prevalence of MetS and its components in males compared to females, with the exception of abdominal obesity which was slightly higher in females [18, 21, 44]. As the average age of menopause is 51 years [45], it is assumed that all females in the current study were postmenopausal. There is a greater risk of abdominal obesity in postmenopausal women, likely related to the decline in estrogen concentration which affects body fat distribution with increasing years post menopause [46], potentially explaining this finding. In addition, socioeconomic deprivation is known to increase the risk of non-communicable diseases [47, 48] and is associated with greater MetS risk [20, 47]. Although a higher proportion of participants with MetS, compared to without, were found to live in the most socioeconomically deprived areas, no association between socioeconomic deprivation and MetS was observed, except in relation to abdominal obesity. This is consistent with our previous findings in the TUDA cohort that greater socioeconomic deprivation was associated with an increase in obesity [27]. In line with previous reports that lower education level is associated with increased MetS risk [18, 21, 49], our participants with MetS were found to have spent fewer years in formal education. The findings thus suggest that older males and those living in more deprived areas and with lower educational attainment are at particular risk of developing MetS.

Few previous studies have examined the relative contribution of specific dietary components in the development and progression of MetS. In the current study, we not only examined macronutrient intakes, but for the first time in a study of this nature we considered protein quality. The findings show that participants with MetS had significantly lower protein intakes, whereas a higher protein intake was found to be protective against MetS, abdominal obesity and hypertension risk, generally consistent with previous reports [50–52]. Of note, the higher protein intake observed to be protective in the current study would equate to 16.1 g protein/day (based on a 70 kg person). In food terms, this is approximately just 2 eggs or 200 g of tofu, thus offering a practical strategy to increasing protein intake. A particularly novel aspect of the current study is that it is the first to investigate protein quality in relation to MetS, as classified here using PDCAAS. Previous studies, albeit not using this method, have examined differences in animal- versus plant-based protein sources with regard to MetS risk. Some such studies report a protective effect on MetS risk of animal protein [50, 52], while others report a protective effect of plant protein [53, 54] or no effect [55]. It was beyond the scope of the current study to examine animal—versus plant-based protein sources; this would have required an extensive re-analysis of the raw dietary data and food sources. It is noteworthy, however, that participants with, compared to without, MetS consumed significantly less high-quality protein foods which, in this cohort, were almost entirely foods of animal origin (with just 2% of the cohort consuming soy products, the only plant source of high-quality protein). Furthermore, participants with MetS consumed significantly more low-quality protein foods which were found to be carbohydrate-rich, low-fiber foods [56]. Within this context, it is worth noting that dietary guidelines for older adults in Ireland recommend a protein-dense diet, including high-quality protein foods, to maintain muscle mass and prevent sarcopenia [56, 57] which is associated with increased risk of mortality [58–60]. Increasing the quantity and quality of protein may also help to maintain bone health and protect against frailty and falls [61, 62]. Thus, the current findings support the position that protein, particularly high-quality protein, should explicitly feature in dietary recommendations and interventions targeting older adults at-risk of MetS.

Apart from protein intakes, the current study found that energy, fiber and PUFA intakes were significantly lower in participants with MetS compared to those without. Furthermore, participants with MetS were found to have higher carbohydrate and free sugar intakes consistent with consuming more carbohydrate-rich, low-fiber foods and lower amounts of protein-rich foods. Previous studies of Korean and Iranian adults [aged 20–69 years] reported higher carbohydrate and lower protein intakes in individuals with MetS [63], and that higher carbohydrate intakes increased MetS risk [64]. In contrast, a lower carbohydrate intake in individuals with MetS was reported in older adults from the Balearic Islands [65]. It is important to note, however, that reducing the intake of one macronutrient will result in an increased intake of one or all other macronutrients [24]. Two studies have examined the effects of macronutrient substitution on MetS risk, with one reporting that substituting carbohydrates for fats or proteins reduced MetS risk [25], but the other found no effect [24]. Apart from protein, MUFA was the only other macronutrient found to be protective against MetS risk in the current study. In addition, participants with MetS had lower dietary fiber intakes. These findings further support the previously reported protective effects of MUFA and fiber in both CVD and MetS risk [66, 67]. As shown elsewhere [68], we found that saturated fat intakes in all participants were above the recommended limit of < 10% EI [69], while free sugar intakes were in line with the < 10% EI recommendation but exceeded the more strict target of < 5% EI [70]. In addition, intakes of DHA and EPA, considered essential for cardiovascular health, were substantially lower than the recommended intake of 250 mg/day [71] in all participants. Our findings therefore suggest that tailored dietary advice promoting adequate and higher quality protein, higher fiber and unsaturated fat intakes is needed, especially for individuals with MetS who are at greatest risk of CVD.

Unsurprisingly, in the current study higher waist circumference and triglyceride concentrations were found to be predictive of MetS development [18, 19, 72]. A higher waist circumference and BMI were associated with increased abdominal obesity risk; however, our finding that HbA1c predicted a lower risk was unexpected given the known associations between blood glucose and abdominal obesity. A higher alcohol intake predicted an increased risk of hypertension, in line with the literature [24], but notably a higher HDL-cholesterol was also predictive of increased hypertension risk. The latter finding may be explained by the beneficial effect of moderate alcohol intake on HDL-cholesterol as previously reported [73]. Although elevated inflammatory markers, such as CRP, IL-6 and TNF-α, have been previously reported in participants with MetS [5], no such relationships were observed in the current study. Finally, while physical activity was not associated with MetS risk, individuals with MetS were found to engage in less exercise in the 2 weeks preceding sampling, supporting the role that physical activity can potentially play in MetS prevention [21].

The findings of the current study have relevance in the development of policy for older adults. The high MetS prevalence, which increases with advancing age, is concerning as it also predisposes to higher risk of CVD and T2DM. Thus, the early detection of MetS is crucial in order to prevent the progression of MetS, and other chronic diseases of aging which pose significant economic burden. The finding that MetS is more prevalent in males than females, and that those living in areas of socioeconomic deprivation are most at-risk of abdominal obesity, emphasizes the need for targeted strategies for at-risk populations. Dietary guidance to promote weight management and ensure good quality protein, optimal unsaturated fat and fiber intakes, as well as guidance on adequate physical activity, should be emphasized in these at-risk groups in particular.

The main strength of this study is that the data are from a large and comprehensively characterized cohort of community-dwelling older adults, recruited from two health jurisdictions in Europe and with follow-up of a sub-set 5.4 years following initial sampling using standardized protocols. Notably, a robust harmonized global definition was used to classify MetS and the availability of data at two timepoints enabled the progression of MetS and contributory factors over time to be examined. Although most baseline characteristics were similar between the total cohort and those who participated in the follow-up study, a potential limitation is that the follow-up sample were slightly younger, better educated and lived in less deprived areas, which may have introduced some bias and could have underestimated the progression of MetS; however, this is unlikely to have changed our main findings. Another limitation is that because fasting blood samples were not collected, the JIS criteria for insulin resistance could not be strictly applied and instead was measured using HbA1c values.

## Conclusion

In conclusion, this study provides novel insights to suggest that enhancing protein quantity and quality may have specific benefits in older people at risk of MetS. Further investigation, in the form of randomized trials, will be required to determine the effect of targeted dietary interventions in delaying the progression of MetS and its components. If confirmed in future trials, the current findings could make a meaningful contribution to the evidence-base to drive nutrition intervention strategies aimed at preventing MetS and its associated pathologies in older people.

### Supplementary Information

Additional file 1.

## Data Availability

Data described in the manuscript, code book, and analytic code will be made available upon request, subject to formal application and approval by the TUDA study consortium.

## Abbreviations

ATPIII: National Cholesterol Education Program Adult Treatment Panel III
CRP: C-reactive protein
CVD: Cardiovascular disease
DHA: Docosahexaenoic acid
DIAAS: Digestible indispensable amino acid score
DRVs: Dietary reference values
EER: Estimated energy requirement
EFSA: European Food Safety Authority
EI: Energy intake
EPA: Eicosapentaenoic acid
FFQ: Food frequency questionnaire
HbA1c: Hemoglobin A1c
HDL-c: High-density lipoprotein cholesterol
IDF: International Diabetes Federation
IL-6: interleukin-6
JIS: Joint Interim Statement
LDL-c: Low-density lipoprotein cholesterol
MetS: Metabolic syndrome
MMSE: Mini-Mental State Examination
MUFA: Monounsaturated fatty acids
PAL: Physical activity level
PDCAAS: Protein digestibility-corrected amino acid score
PSMS: Physical self-maintenance scale
PUFA: Polyunsaturated fatty acids
RDA: Recommended dietary allowance
T2DM: Type 2 diabetes mellitus
TIA: Transient ischemic attack
TNF-α: Tumor necrosis factor-alpha
TUDA: Trinity-Ulster-Department of Agriculture
TUG: Timed up-and-go
