# Supplementary material for: Identification of nutrition factors in the metabolic syndrome and its progression over time in older adults: analysis of the TUDA cohort
Source: Diabetol Metab Syndr. 2024 Jun 8;16:125. doi: 10.1186/s13098-024-01367-z (PMC11162058; doi:10.1186/s13098-024-01367-z)
Supplement: Supplementary file 1 — Additional file 1. [file 13098_2024_1367_MOESM1_ESM.docx]

**Additional file 1.**

**Figure S1.** Directed acyclic graph of the hypothesized relationships between MetS, diet and the covariates.

**
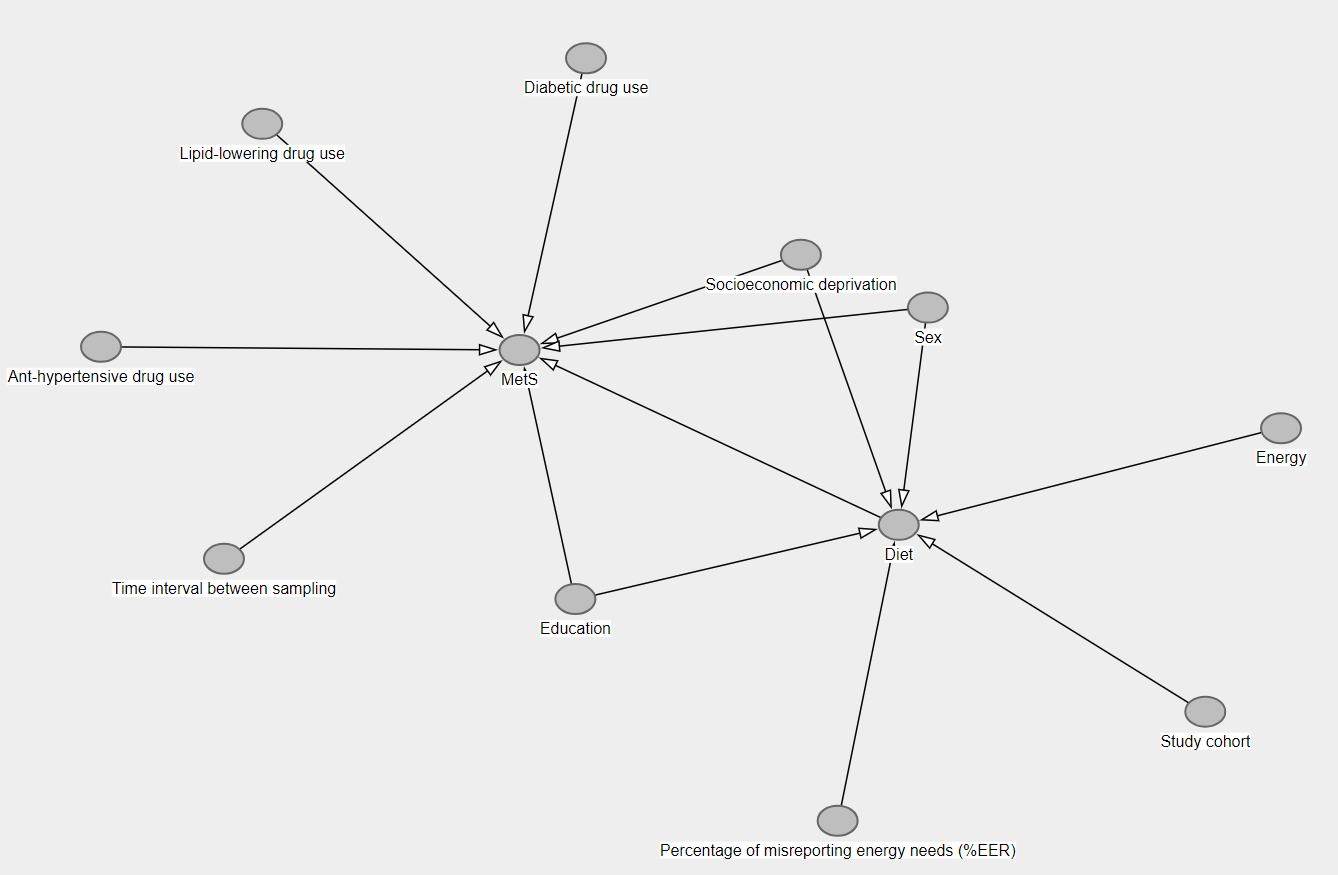
**

**Table S1.** Baseline characteristics of the total available TUDA cohort and the follow-up sample

|  | Baseline sample | |  |
| --- | --- | --- | --- |
|  | *n* 3,487 | *n* 953 | *P*^1^ |
| Age (years) | 70.5 (69.8, 71.1) | 68.9 (66.6, 71.1) | **<0.001** |
| Male sex, *n* (%) | 1138 (33) | 317 (33) | 0.628 |
| Age formal education ended, *n* (%) |  |  |  |
| ≤14 | 1285 (37) | 304 (32) | **<0.001** |
| 15–16 | 1059 (30) | 247 (26) | **<0.001** |
| 17–18 | 525 (15) | 165 (17) | **0.023** |
| ≥19 | 613 (18) | 236 (25) | **<0.001** |
| Socioeconomic deprivation, *n* (%)^2^ |  |  |  |
| Less deprived (Q1, Q2, Q3) | 1988 (57) | 633 (66) | **<0.001** |
| More deprived (Q4, Q5) | 1361 (39) | 288 (30) | **<0.001** |
| *MetS^3^ components and related factors* |  |  |  |
| Waist circumference (cm) | 95.8 (95.3, 96.2) | 95.2 (94.3, 96.1) | **0.042** |
| Triglycerides (mmol/L) | 1.7 (1.6, 1.7) | 1.6 (1.6, 1.7) | 0.201 |
| HDL cholesterol (mmol/L) | 1.5 (1.5, 1.5) | 1.6 (1.5, 1.5) | 0.064 |
| LDL cholesterol (mmol/L) | 2.5 (2.5, 2.5) | 2.6 (2.5, 2.6) | 0.064 |
| Systolic BP (mmHg) | 144.8 (144.1, 145.5) | 143.7 (142.4, 144.9) | 0.108 |
| Diastolic BP (mmHg) | 79.1 (78.7, 79.4) | 78.6 (78.0, 79.3) | **0.043** |
| Hypertensive, *n* (%)^4^ | 2047 (59) | 540 (57) | 0.129 |
| HbA1c (mmol/mol)^5^ | 40.6 (40.3, 40.9) | 39.9 (39.4, 40.4) | **0.028** |
| Normoglycemic, *n* (%) | 1644 (47) | 482 (51) | **0.015** |
| Hyperglycemic, *n* (%) | 1689 (48) | 432 (45) | **0.015** |
| Prediabetic, *n* (%) | 1293 (77) | 337 (78) | 0.161 |
| Diabetic, *n* (%) | 396 (23) | 95 (22) | 0.103 |
| *Other health and lifestyle factors* |  |  |  |
| Waist-to-hip ratio (cm) | 0.91 (0.91, 0.91) | 0.90 (0.90, 0.91) | **<0.001** |
| Weight (kg) | 74.9 (74.3, 75.4) | 75.9 (74.8, 76.9) | 0.149 |
| Height (m) | 1.62 (1.62, 1.63) | 1.64 (1.63, 1.64) | **<0.001** |
| BMI (kg/m^2^)^6^ | 28.3 (28.2, 28.5) | 28.2 (27.9, 28.5) | 0.248 |
| Overweight, *n* (%) | 1389 (40) | 389 (41) | 0.476 |
| Obese, *n* (%) | 1144 (33) | 297 (31) | 0.199 |
| Timed Up-and-Go (seconds)^7^ | 10.1 (10.0, 10.3) | 9.3 (9.1, 9.6) | **<0.001** |
| Physical self-maintenance scale score^8^ | 23.3 (23.3, 23.4) | 23.5 (23.4, 23.6) | **<0.001** |
| Physical activity, *n* (%)^9^ | 2866 (82) | 827 (87) | **<0.001** |
| Living alone, *n* (%) | 990 (28) | 236 (25) | **0.004** |
| Current smoker, *n* (%) | 432 (12) | 89 (9) | **<0.001** |
| Past smoker, *n* (%) | 3054 (88) | 863 (91) | **<0.001** |
| Alcohol (units/week)^10^ | 8.4 (7.8, 8.9) | 8.0 (7.1, 8.9) | 0.213 |
| Fortified food consumer, *n* (%)^11^ | 2481 (71) | 681 (72) | 0.784 |
| *Self-reported medical history* |  |  |  |
| Diabetes, *n* (%) | 425 (12) | 95 (10) | **0.049** |
| Hyperlipidemia, *n* (%) | 1967 (56) | 541 (57) | **0.010** |
| Previous myocardial infarction, *n* (%) | 309 (9) | 77 (8) | 0.593 |
| Previous TIA, *n* (%) | 203 (6) | 51 (5) | 0.754 |
| Previous stroke, *n* (%) | 105 (3) | 16 (2) | **0.018** |

Data expressed as mean (95% CI), except where stated otherwise. This study involved new analysis of existing samples from the Trinity-Ulster-Department of Agriculture (TUDA) cohort (*n* 3,487) first sampled in 2008–2012 for comprehensive health, but not dietary, data. The TUDA follow-up sample comprises about 20% of the original cohort who were followed up for re-investigation in 2014–2018 (*n* 953).

^1^Differences in continuous variables were analyzed by ANCOVA (adjusting for age and sex) on log-transformed data as appropriate with Bonferroni post-hoc tests. Categorical variables were analyzed by chi-square. *P*<0.05 was considered significant; significant values are highlighted in bold text.

^2^Area-based socioeconomic deprivation score from individual geo-referenced address-based information, as previously described (27). Deprivation scores were categorized into quintiles (Q1–5), with Q1 being the 20% least deprived category, and Q5 the 20% most deprived category. For this analysis, participants in Q1, Q2 and Q3 were grouped into ‘less deprived’ and participants in Q4 and Q5 were grouped into ‘more deprived’.

^3^MetS is a clustering of abnormal metabolic components including abdominal obesity, elevated blood pressure, reduced HDL cholesterol, elevated triglycerides and impaired fasting glucose.

^4^Defined as systolic blood pressure (BP) ≥140 mmHg and/or diastolic BP ≥90 mmHg (72, 73).

^5^HbA1c was used to define participants as normoglycemic (<39 mmol/mol); hyperglycemic (≥39 mmol/mol); prediabetic (≥39 to ≤47 mmol/mol); and diabetic (≥48 mmol/mol) (38).

^6^World Health Organization BMI cut-offs (74): overweight (≥25 to ≤29.9 kg/m^2^) and obesity (≥30 kg/m^2^). Of note, *n* 58 (2%) of the baseline sample and *n* 16 (2%) of the follow-up sample were identified as underweight (<18.5 kg/m^2^), while *n* 829 (24%) of the baseline sample and *n* 244 (26%) of the follow-up sample were identified as normal weight (≥18.5 to ≤24.9 kg/m^2^).

^7^Timed Up-and-Go test measured the time taken to stand up from seated in a chair, walk three meters, turn around and walk back to return to the original seated position.

^8^The physical self-maintenance scale questionnaire assigns scores to the participants highest level of functioning for activities of daily living, the higher the total score the more independent the participant.

^9^Any exercise in the last two weeks.

^10^Alcohol units per week among those consuming alcohol: *n* 1531 (60%) of the participants recruited at baseline only; *n* 634 (67%) of the participants recruited at both baseline and follow-up. One unit equates with 25 mL spirits, 220 mL beer, and 85 mL wine.

^11^Participants who consumed foods fortified with B-vitamins at least once per week.

Abbreviations: BMI, body mass index; HbA1c, hemoglobin A1c; HDL, high-density lipoprotein; LDL, low-density lipoprotein; MetS, metabolic syndrome; TIA, transient ischemic attack.

**Table S2.** Nutrition-related factors and metabolic syndrome (MetS)^1^ characteristics of TUDA participants who had MetS at baseline but not at follow-up

|  | TUDA participants | |  |
| --- | --- | --- | --- |
|  | Baseline  (*n* 76) | Follow-up  (*n* 76) | *P* value |
| Age (years) | 69.6 (68.3, 70.9) | 75.7 (74.6, 76.8) | **<0.001** |
| Male sex, *n* (%) | 25 (33) | 25 (33) | - |
| Age formal education ended (years) | 17.3 (16.5, 18.0) | 17.3 (16.5, 18.0) | - |
| Socioeconomic deprivation, *n* (%)^2^ |  |  |  |
| Less deprived (Q1, Q2, Q3) | 50 (66) | 50 (66) | - |
| More deprived (Q4, Q5) | 23 (30) | 23 (30) | - |
| *Drug treatments* |  |  |  |
| Lipid-lowering drugs*, n* (%) | 35 (46) | 19 (25) | **<0.001** |
| Anti-hypertensive drugs, *n* (%) | 48 (63) | 46 (61) | 0.752 |
| Diabetic drugs*, n* (%) | 2 (3) | 2 (3) | 1.000 |
| *MetS components and related factors* |  |  |  |
| Waist circumference (cm) | 96.5 (93.4, 99.5) | 96.0 (92.9, 99.2) | 0.749 |
| Triglycerides (mmol/L) | 1.6 (1.5, 1.7) | 1.4 (1.3, 1.5) | **0.017** |
| HDL cholesterol (mmol/L) | 1.4 (1.3, 1.5) | 1.6 (1.5, 1.8) | **<0.001** |
| LDL cholesterol (mmol/L) | 2.8 (2.5, 3.0) | 2.5 (2.3, 2.7) | **0.002** |
| Systolic BP (mmHg) | 144.0 (139.3, 148.7) | 136.7 (131.0, 142.4) | **0.029** |
| Diastolic BP (mmHg) | 79.7 (77.4, 82.0) | 80.6 (78.0, 83.2) | 0.534 |
| Hypertensive, *n* (%)^3^ | 47 (62) | 34 (45) | **0.012** |
| HbA1c (mmol/mol)^4^ | 38.7 (37.6, 39.9) | 37.9 (36.4, 39.5) | 0.086 |
| Normoglycemic, *n* (%) | 35 (46) | 60 (79) | **<0.001** |
| Hyperglycemic, *n* (%) | 36 (47) | 14 (18) | **<0.001** |
| Prediabetic, *n* (%) | 4 (11) | 11 (79) | **<0.001** |
| Diabetic, *n* (%) | 32 (89) | 3 (21) | **<0.001** |
| *Other health and lifestyle factors* |  |  |  |
| Waist-to-hip ratio (cm) | 0.90 (0.89, 0.92) | 0.92 (0.90, 0.93) | 0.129 |
| Weight (kg) | 77.5 (73.4, 81.6) | 74.4 (70.2, 78.5) | **<0.001** |
| Height (m) | 1.63 (1.61, 1.66) | 1.62 (1.60, 1.65) | **<0.001** |
| BMI (kg/m^2^)^5^ | 28.9 (27.7, 30.2) | 28.2 (26.9, 29.5) | **0.005** |
| Overweight, *n* (%) | 32 (42) | 27 (36) | 0.502 |
| Obese, *n* (%) | 28 (37) | 23 (30) | 0.182 |
| Timed Up-and-Go (seconds)^6^ | 9.0 (8.3, 9.7) | 10.9 (10.2, 11.6) | **<0.001** |
| Physical self-maintenance scale score^7^ | 23.5 (23.3, 23.8) | 23.2 (22.9, 23.6) | 0.090 |
| Physical activity, *n* (%)^8^ | 68 (90) | 64 (84) | 0.386 |
| Living alone, *n* (%) | 13 (17) | 17 (22) | 0.221 |
| Current smoker, *n* (%) | 4 (5) | 4 (5) | 1.000 |
| Past smoker, *n* (%) | 33 (43) | 35 (46) | 0.617 |
| Alcohol (units/week)^9^ | 9.0 (3.8, 14.1) | 5.1 (3.1, 7.0) | 0.866 |
| Fortified food consumer, *n* (%)^10^ | 53 (70) | 45 (59) | 0.201 |
| *Self-reported medical history* |  |  |  |
| Diabetes, *n* (%) | 2 (3) | 7 (9) | 0.074 |
| Hyperlipidemia, *n* (%) | 34 (45) | 30 (40) | 0.663 |
| Previous myocardial infarction, *n* (%) | 0 (0) | 0 (0) | - |
| Previous transient ischemic attack, *n* (%) | 5 (7) | 6 (8) | 1.000 |
| Previous stroke, *n* (%) | 1 (1) | 2 (3) | 1.000 |

Data expressed as mean (95% CI), except where stated otherwise. Data obtained from the Trinity-Ulster-Department of Agriculture (TUDA) baseline sample (2008–2012) and the corresponding follow-up sample (2014–2018). Continuous variables were analyzed using paired samples t-tests on log-transformed data. Categorical variables were analyzed using McNemar’s test. *P*<0.05 was considered significant; significant values are highlighted in bold text.

^1^Participants were deemed to have MetS if they met at least three of the following criteria: waist circumference of ≥102 cm or ≥88 cm, for males and females respectively (37); elevated triglycerides of ≥1.7 mmol/L (≥150 mg/dL) (4); reduced HDL cholesterol of <1.0 mmol/L (<40 mg/dL) for males and <1.3 mmol/L (<50 mg/dL) for females (4); elevated blood pressure of systolic ≥130 and/or diastolic ≥85 mmHg (4); and HbA1c of ≥39 mmol/mol (38).

^2^Area-based socioeconomic deprivation score from individual geo-referenced address-based information, as previously described (27). Deprivation scores were categorized into quintiles (Q1–5), with Q1 being the 20% least deprived category, and Q5 the 20% most deprived category. For this analysis, participants in Q1, Q2 and Q3 were grouped into ‘less deprived’ and participants in Q4 and Q5 were grouped into ‘more deprived’.

^3^Defined as systolic blood pressure (BP) ≥140 mmHg and/or diastolic BP ≥90 mmHg (72, 73).

^4^HbA1c was used to define participants as normoglycemic (<39 mmol/mol); hyperglycemic (≥39 mmol/mol); prediabetic (≥39 to ≤47 mmol/mol); and diabetic (≥48 mmol/mol) (38).

^5^World Health Organization BMI cut-offs (74): overweight (≥25 to ≤29.9 kg/m^2^) and obesity (≥30 kg/m^2^).

^6^Timed Up-and-Go test measured the time taken to stand up from seated in a chair, walk three meters, turn around and walk back to return to the original seated position.

^7^The physical self-maintenance scale is a questionnaire which assigns scores to the participants highest level of functioning for activities of daily living, the higher the total score the more independent the participant.

^8^Any exercise in the last two weeks.

^9^Alcohol units per week among those consuming alcohol: *n* 55 (72%) of the baseline sample; *n* 54 (71%) of the follow-up sample. One unit equates with 25 mL spirits, 220 mL beer, and 85 mL wine.

^10^Participants who consumed foods fortified with B-vitamins at least once per week.

Abbreviations: BMI, body mass index; HbA1c, hemoglobin A1c; HDL, high-density lipoprotein; LDL, low-density lipoprotein; TIA, transient ischemic attack.

**Table S3.** Nutrition-related factors and metabolic syndrome (MetS)^1^ characteristics in male and female participants at follow-up

|  | Males | | | Females | | |
| --- | --- | --- | --- | --- | --- | --- |
|  | With MetS  (*n* 260) | Without MetS  (*n* 57) | *P* value | With MetS  (*n* 445) | Without MetS  (*n* 191) | *P* value |
| Age (years) | 76.2 (75.6, 76.8) | 76.1 (74.9, 77.3) | 0.915 | 76.1 (75.6, 76.6) | 74.6 (73.9, 75.3) | **<0.001** |
| Age formal education ended, *n* (%) |  |  |  |  |  |  |
| ≤14 | 88 (34) | 18 (31) | 0.681 | 151 (34) | 47 (25) | **0.022** |
| 15–16 | 81 (31) | 11 (19) | 0.064 | 117 (26) | 38 (20) | 0.091 |
| 17–18 | 33 (13) | 9 (16) | 0.566 | 76 (17) | 44 (23) | 0.073 |
| ≥19 | 58 (22) | 20 (35) | 0.051 | 99 (22) | 59 (31) | **0.019** |
| Socioeconomic deprivation, *n* (%)^2^ |  |  |  |  |  |  |
| Less deprived (Q1, Q2, Q3) | 184 (71) | 41 (71) | 0.957 | 278 (63) | 130 (68) | 0.113 |
| More deprived (Q4, Q5) | 64 (25) | 14 (24) | 0.957 | 156 (35) | 54 (28) | 0.113 |
| *Drug treatments* |  |  |  |  |  |  |
| Lipid-lowering drugs*, n* (%) | 210 (81) | 7 (12) | **<0.001** | 340 (76) | 23 (12) | **<0.001** |
| Anti-hypertensive drugs, *n* (%) | 247 (95) | 46 (79) | **<0.001** | 368 (83) | 87 (46) | **<0.001** |
| Diabetic drugs*, n* (%) | 51 (20) | 0 (0) | **<0.001** | 50 (11) | 4 (2) | **<0.001** |
| *MetS components and related factors* |  |  |  |  |  |  |
| Waist circumference (cm) | 106.5 (105.2, 107.9) | 98.0 (95.4, 100.5) | **<0.001** | 97.1 (95.8, 98.4) | 86.7 (85.0, 88.5) | **<0.001** |
| Triglycerides (mmol/L) | 1.7 (1.6, 1.9) | 1.4 (1.3, 1.6) | 0.070 | 1.8 (1.5, 1.6) | 1.2 (1.2, 1.3) | **<0.001** |
| HDL cholesterol (mmol/L) | 1.3 (1.2, 1.3) | 1.5 (1.4, 1.6) | **<0.001** | 1.7 (1.6, 1.7) | 2.0 (1.9, 2.0) | **<0.001** |
| LDL cholesterol (mmol/L) | 1.8 (1.7, 1.9) | 2.6 (2.3, 2.8) | **<0.001** | 2.2 (2.1, 2.3) | 2.8 (2.7, 2.9) | **<0.001** |
| Systolic BP (mmHg) | 146.0 (143.5, 148.6) | 143.6 (138.6, 148.5) | 0.467 | 140.6 (138.7, 142.5) | 134.3 (131.1, 137.6) | **0.002** |
| Diastolic BP (mmHg) | 79.4 (78.1, 80.7) | 82.7 (79.6, 85.8) | **0.045** | 80.2 (79.1, 81.3) | 81.5 (79.9, 83.0) | 0.441 |
| Hypertensive, *n* (%)^3^ | 157 (60) | 36 (62) | 0.812 | 232 (52) | 76 (40) | **0.008** |
| HbA1c (mmol/mol)^4^ | 43.7 (42.5, 44.9) | 37.3 (36.1, 38.4) | **<0.001** | 41.7 (40.8, 42.5) | 36.6 (35.9, 37.4) | **<0.001** |
| Normoglycemic, *n* (%) | 83 (32) | 46 (79) | **<0.001** | 161 (36) | 155 (82) | **<0.001** |
| Hyperglycemic, *n* (%) | 176 (68) | 11 (19) | **<0.001** | 278 (63) | 29 (15) | **<0.001** |
| Prediabetic, *n* (%) | 114 (65) | 10 (91) | **<0.001** | 214 (77) | 25 (86) | **<0.001** |
| Diabetic, *n* (%) | 62 (35) | 1 (9) | **<0.001** | 64 (23) | 4 (14) | **<0.001** |
| *Other health and lifestyle factors* |  |  |  |  |  |  |
| Waist-to-hip ratio (cm) | 0.98 (0.97, 0.99) | 0.95 (0.94, 0.97) | **<0.001** | 0.91 (0.91, 0.92) | 0.87 (0.86, 0.88) | **<0.001** |
| Weight (kg) | 88.4 (86.6, 90.2) | 79.0 (75.1, 82.8) | **<0.001** | 71.1 (69.7, 72.4) | 62.0 (60.2, 63.8) | **<0.001** |
| Height (m) | 1.71 (1.70, 1.74) | 1.73 (1.72, 1.74) | 0.209 | 1.57 (1.56, 1.58) | 1.57 (1.57, 1.58) | 0.822 |
| BMI (kg/m^2^)^5^ | 29.4 (28.9, 29.9) | 26.7 (25.5, 27.8) | **<0.001** | 28.8 (28.2, 29.3) | 25.0 (24.3, 25.7) | **<0.001** |
| Overweight, *n* (%) | 112 (43) | 28 (48) | 0.471 | 188 (42) | 57 (30) | **0.005** |
| Obese, *n* (%) | 107 (41) | 10 (17) | **<0.001** | 147 (33) | 22 (12) | **<0.001** |
| Timed Up-and-Go (seconds)^6^ | 10.6 (10.2, 11.0) | 10.9 (9.4, 12.4) | 0.932 | 12.6 (11.9, 13.3) | 10.9 (10.4, 11.3) | **0.008** |
| Physical self-maintenance scale score^7^ | 23.6 (23.5, 23.7) | 23.5 (23.3, 23.8) | 0.815 | 22.8 (22.6, 23.0) | 23.5 (23.3, 23.7) | **<0.001** |
| Physical activity, *n* (%)^8^ | 219 (84) | 50 (86) | 0.706 | 375 (84) | 179 (94) | **<0.001** |
| Living alone, *n* (%) | 48 (19) | 10 (17) | 0.902 | 176 (40) | 60 (32) | 0.200 |
| Current smoker, *n* (%) | 15 (6) | 1 (2) | 0.203 | 24 (5) | 15 (8) | 0.229 |
| Past smoker, *n* (%) | 157 (60) | 36 (62) | 0.812 | 160 (36) | 69 (36) | 0.931 |
| Alcohol (units/week)^9^ | 7.8 (6.3, 9.2) | 8.7 (6.3, 11.1) | 0.619 | 2.6 (2.2, 3.0) | 3.4 (2.6, 4.2) | 0.205 |
| Fortified food consumer, *n* (%)^10^ | 147 (57) | 29 (50) | 0.365 | 300 (67) | 127 (67) | 0.888 |
| *Self-reported medical history* |  |  |  |  |  |  |
| Diabetes, *n* (%) | 65 (25) | 3 (5) | **0.002** | 60 (14) | 7 (4) | **<0.001** |
| Hyperlipidemia, *n* (%) | 157 (60) | 12 (21) | **<0.001** | 328 (74) | 60 (32) | **<0.001** |
| Previous myocardial infarction, *n* (%) | 53 (20) | 0 (0) | **<0.001** | 34 (8) | 3 (2) | **0.005** |
| Previous transient ischemic attack, *n* (%) | 27 (10) | 2 (3) | 0.242 | 44 (10) | 8 (4) | 0.053 |
| Previous stroke, *n* (%) | 9 (4) | 1 (2) | 0.627 | 13 (3) | 2 (1) | 0.061 |

Data expressed as mean (95% CI), except where stated otherwise. Data obtained from the Trinity-Ulster-Department of Agriculture (TUDA) follow-up sample (*n* 953). Continuous variables were analyzed using ANCOVA (adjusting for age) on log-transformed data as appropriate with Bonferroni post-hoc tests. Categorical variables were analyzed using chi-square. *P*<0.05 was considered significant; significant values are highlighted in bold text.

^1^Participants were deemed to have MetS if they met at least three of the following criteria: waist circumference of ≥102 cm or ≥88 cm, for males and females respectively (37); elevated triglycerides of ≥1.7 mmol/L (≥150 mg/dL) (4); reduced HDL cholesterol of <1.0 mmol/L (<40 mg/dL) for males and <1.3 mmol/L (<50 mg/dL) for females (4); elevated blood pressure of systolic ≥130 and/or diastolic ≥85 mmHg (4); and HbA1c of ≥39 mmol/mol (38).

^2^Area-based socioeconomic deprivation score from individual geo-referenced address-based information, as previously described (27). Deprivation scores were categorized into quintiles (Q1–5), with Q1 being the 20% least deprived category, and Q5 the 20% most deprived category. For this analysis, participants in Q1, Q2 and Q3 were grouped into ‘less deprived’ and participants in Q4 and Q5 were grouped into ‘more deprived’.

^3^Defined as systolic blood pressure (BP) ≥140 mmHg and/or diastolic BP ≥90 mmHg (72, 73).

^4^HbA1c was to define participants as normoglycemic (<39 mmol/mol); hyperglycemic (≥39 mmol/mol); prediabetic (≥39 to ≤47 mmol/mol); and diabetic (≥48 mmol/mol) (38).

^5^World Health Organization BMI cut-offs (74): overweight (≥25 to ≤29.9 kg/m^2^) and obesity (≥30 kg/m^2^). Of note, *n* 0 (0%) males with and without MetS, *n* 7 (2%) females with MetS and *n* 9 (5%) females without MetS were identified as underweight (<18.5 kg/m^2^), while *n* 37 (14%) males with MetS, *n* 20 (35%) males without MetS, *n* 90 (20%) females with MetS and *n* 97 (51%) females without MetS were identified as normal weight (≥18.5 to ≤24.9 kg/m^2^).

^6^Timed Up-and-Go test measured the time taken to stand up from seated in a chair, walk three meters, turn around and walk back to return to the original seated position

^7^The physical self-maintenance scale is a questionnaire which assigns scores to the participants highest level of functioning for activities of daily living, the higher the total score the more independent the participant.

^8^Any exercise in the last two weeks.

^9^Alcohol units per week among those consuming alcohol: *n* 176 (68%) and *n* 50 (86%) of males with and without MetS, respectively; *n* 247 (56%) and *n* 129 (68%) of females with and without MetS, respectively. One unit equates with 25 mL spirits, 220 mL beer, and 85 mL wine.

^10^Participants who consumed foods fortified with B-vitamins at least once per week.

Abbreviations: BMI, body mass index; HbA1c, hemoglobin A1c; HDL, high-density lipoprotein; LDL, low-density lipoprotein.

**Table S4.** Daily energy and macronutrient intakes of Irish older adults with and without metabolic syndrome (MetS)^1^

|  | Males | |  |  | Females | |  |  |
| --- | --- | --- | --- | --- | --- | --- | --- | --- |
|  | With MetS  (*n* 229) | Without MetS  (*n* 51) | *P* value | DRVs^2^ | With MetS  (*n* 367) | Without MetS  (*n* 156) | *P* value | DRVs^2^ |
| Energy (MJ) | 7.88 (2.57) | 7.77 (3.29) | **0.005** | 8.4–11.9 | 6.75 (2.10) | 7.16 (2.17) | **<0.001** | 6.8–9.6 |
| Energy (kcal)^3^ | 1873 (616) | 1848 (793) | **0.006** | 2017–2834 | 1607 (506) | 1706 (512) | **<0.001** | 1628–2305 |
| Protein (g) | 80.2 (20.7) | 84.1 (27.7) | 0.066 | – | 68.6 (21.4) | 74.4 (20.6) | **0.031** | – |
| Protein (%EI) | 16.5 (4.3) | 17.2 (3.8) | **0.039** | – | 17.1 (4.0) | 17.3 (4.3) | **0.032** | – |
| Protein (g/kg bw) | 0.90 (0.33) | 1.06 (0.39) | **0.002** | 0.83 | 0.99 (0.42) | 1.2 (0.42) | **0.002** | 0.83 |
| Total Fat (g) | 70.4 (29.1) | 74.8 (31.7) | 0.654 | – | 62.1 (27.7) | 69.2 (25.5) | 0.354 | – |
| Total Fat (%EI) | 34.5 (7.2) | 34.5 (6.3) | 0.603 | 20–35 | 35.1 (6.8) | 35.3 (6.9) | 0.319 | 20–35 |
| Saturated fat (g) | 27.8 (15.6) | 27.3 (14.8) | 0.745 | – | 23.5 (11.9) | 24.4 (13.9) | 0.237 | – |
| Saturated Fat (%EI) | 13.4 (4.7) | 13.2 (3.7) | 0.776 | ≤10% EI^4^ | 13.5 (4.3) | 13.4 (4.5) | 0.232 | ≤10% EI^4^ |
| MUFA (g) | 24.2 (11.2) | 26.5 (11.3) | 0.257 | – | 20.6 (9.4) | 23.2 (9.5) | 0.170 | – |
| MUFA (%EI) | 11.5 (3.1) | 11.9 (3.6) | 0.232 | – | 11.7 (2.9) | 12.0 (3.0) | 0.172 | – |
| PUFA (g) | 9.3 (5.7) | 10.5 (4.5) | 0.634 | – | 8.5 (4.5) | 9.8 (5.3) | **0.043** | – |
| PUFA (%EI) | 4.6 (2.1) | 4.6 (2.2) | 0.415 | – | 4.8 (2.2) | 5.0 (2.5) | **0.024** | – |
| DHA+EPA (mg) | 42.2 (42.0) | 40.0 (50.0) | 0.559 | 250 | 31.0 (30.0) | 35.7 (43.0) | 0.202 | 250 |
| Carbohydrate (g) | 217.5 (76.4) | 200.7 (102.8) | **0.032** | – | 187.5 (64.4) | 187.0 (76.0) | **0.001** | – |
| Carbohydrate (%EI) | 46.9 (7.6) | 43.9 (9.8) | **0.029** | 45–60 | 47.2 (7.4) | 45.3 (8.7) | **<0.001** | 45–60 |
| Starch (g) | 120.4 (48.6) | 110.9 (54.7) | **0.013** | – | 94.1 (34.9) | 94.0 (42.1) | **0.012** | – |
| Total Sugar (g) | 87.2 (43.1) | 87.3 (48.0) | 0.911 | – | 79.3 (39.0) | 84.3 (41.0) | 0.305 | – |
| Free Sugar (g) | 36.9 (31.7) | 35.8 (43.3) | 0.193 | ALAP | 29.2 (25.7) | 29.8 (28.2) | 0.111 | ALAP |
| Free Sugar (%EI) | 7.9 (6.0) | 7.0 (8.0) | 0.134 | <10% EI^5^  <5% EI^5^ | 7.2 (5.8) | 7.3 (5.4) | 0.075 | <10% EI^5^  <5% EI^5^ |
| Fiber (g) | 20.0 (8.7) | 22.3 (8.3) | 0.106 | 25 | 18.1 (7.1) | 19.3 (8.0) | 0.193 | 25 |

Data expressed as median (IQR). Dietary data obtained from the Trinity-Ulster-Department of Agriculture (TUDA) follow-up sample (*n* 803). Variables were analyzed by ANCOVA (adjusting for energy, sex and percentage of misreporting of energy needs (%EER)) on log-transformed data as appropriate with Bonferroni post-hoc tests. *P*<0.05 was considered significant; significant values are highlighted in bold text.

^1^Participants were deemed to have MetS if they met at least three of the following criteria: waist circumference of ≥102 cm or ≥88 cm, for males and females respectively (37); elevated triglycerides of ≥1.7 mmol/L (≥150 mg/dL) (4); reduced HDL cholesterol of <1.0 mmol/L (<40 mg/dL) for males and <1.3 mmol/L (<50 mg/dL) for females (4); elevated blood pressure of systolic ≥130 and/or diastolic ≥85 mmHg (4); and HbA1c of ≥39 mmol/mol (38).

^2^European Food Safety Authority (EFSA) Dietary Reference Values (DRVs) for energy and each macronutrient, where applicable (69).

^3^Of note, 23% of participants with MetS (27% males, 20% females) and 13% of participants without MetS (23% males, 10% females) were identified as potential mis-reporters. Potential misreporting was estimated using predicted values for basal metabolic rate (Oxford equations) (31) and physical activity levels (32). Potential mis-reporters were not excluded from analysis.

^4^World Health Organization strong recommendation (67).

^5^Free sugar limits of <10% energy and <5% energy were derived from WHO guidelines (68).

Abbreviations: %EI, % energy intake; ALAP, as low as possible; bw, body weight; DHA, docosahexaenoic acid; DRVs, dietary reference values; EPA, eicosapentaenoic acid; HbA1c, hemoglobin A1c; MUFA, monounsaturated fatty acid; PUFA, polyunsaturated fatty acid.

**Table S5.** Food groups contributing (%) to protein intake in males and females, with and without metabolic syndrome^1^

|  | Males | | Females | |
| --- | --- | --- | --- | --- |
|  | With MetS  (*n* 229) | Without MetS  (*n* 51) | With MetS  (*n* 367) | Without MetS  (*n* 156) |
| Meat and meat products | 36 | 36 | 33 | 34 |
| Milk and yoghurt | 14 | 14 | 17 | 17 |
| Fish and fish dishes | 7 | 11 | 8 | 9 |
| Bread and rolls | 13 | 11 | 11 | 10 |
| Eggs and egg dishes | 4 | 4 | 4 | 4 |
| Potatoes and potato products | 5 | 4 | 4 | 4 |
| Vegetables and vegetable dishes | 4 | 4 | 5 | 4 |
| Grains, rice, pasta and savories | 4 | 4 | 4 | 3 |
| Biscuits, cakes and pastries | 5 | 4 | 5 | 3 |

Dietary data from the Trinity-Ulster-Department of Agriculture (TUDA) follow-up sample, available for *n* 803. Data is presented as the percentage contribution of the food group to total protein intake.

^1^MetS is a clustering of abnormal metabolic components including abdominal obesity, elevated blood pressure, reduced high-density lipoprotein (HDL) cholesterol, elevated triglycerides and impaired fasting glucose.

**Figure S2.** Protein intake (% energy intake) from the four protein quality food categories^1^ in participants living in the most and least deprived socioeconomic areas^2^ at follow-up.

*P* = 0.662

*P* = 0.058

*P* = 0.093

*P* = 0.970

Dietary data from the Trinity-Ulster-Department of Agriculture (TUDA) follow-up sample, available for *n* 803. Differences between groups were analyzed by independent samples t-test on log-transformed data; *P*<0.05 was considered significant.

^1^Protein quality was assessed using the protein digestibility-corrected amino acid score (PDCAAS). The higher the PDCAAS, the better the quality of the protein. The protein quality categories were defined as follows: category 1 (PDCAAS >95), category 2 (PDCASS 80–90), category 3 (PDCAAS 60–70) and category 4 (PDCAAS <35).

^2^Area-based socioeconomic deprivation score from individual geo-referenced address-based information, as previously described (27). Deprivation scores were categorized into quintiles (Q1–5), with Q1 being the 20% least deprived category, and Q5 the 20% most deprived category. For this analysis, participants in Q1, Q2 and Q3 were grouped into ‘least deprived’ and participants in Q4 and Q5 were grouped into ‘most deprived’.
